# Internet- and mobile-based aftercare and relapse prevention interventions for anxiety and depressive disorders: a systematic review

**DOI:** 10.3389/fpsyg.2024.1474016

**Published:** 2024-12-12

**Authors:** Ligiana Mihaela Petre, Paweł Adam Piepiora, Maria Gemescu, Delia Alexandra Gheorghe

**Affiliations:** ^1^Laboratory of Advanced Studies in Clinical Psychology, Faculty of Psychology and Educational Sciences, University of Bucharest, Bucharest, Romania; ^2^Faculty of Physical Education and Sports, Wroclaw University of Health and Sport Sciences, Wrocław, Poland; ^3^Faculty of Psychology and Educational Sciences, University of Bucharest, Bucharest, Romania; ^4^Department of Experimental and Theoretical Neuroscience, Transylvanian Institute of Neuroscience, Cluj-Napoca, Romania

**Keywords:** depression, anxiety, digital interventions, aftercare, relapse prevention, internet-based, mobile-based, systematic review

## Abstract

**Background:**

Digital interventions present potential solutions for aftercare and relapse prevention in anxiety and depressive disorders. This systematic review synthesizes evidence on the efficacy of internet- and mobile-based interventions for post-acute care in these conditions.

**Methods:**

A systematic search was conducted in electronic databases (MEDLINE, CENTRAL, Scopus, Web of Science, PsycINFO, PsycARTICLES, PsycEXTRA, ProQuest Dissertations and Theses Open, Open Access Theses and Dissertations, and Open Grey) for randomized controlled trials evaluating digital aftercare or relapse prevention interventions for adults with anxiety or depressive disorders. Primary outcomes included symptom severity, relapse rates, recurrence rates, and rehospitalization. Secondary outcomes included general quality of life and adherence to primary treatment. Risk of bias was assessed using the Cochrane tool.

**Results:**

Nineteen studies (3,206 participants) met the inclusion criteria. Interventions included cognitive-behavioral therapy, mindfulness-based approaches, and supportive text messaging. Most studies focused on depression, with limited evidence for anxiety disorders. Notably, fourteen studies that reported on depressive symptoms demonstrated significant improvements following digital interventions, with effect sizes ranging from small (Cohen’s *d* = 0.20) to large (Cohen’s *d* = 0.80). Five studies investigated relapse or recurrence rates, yielding mixed results. Adherence rates varied significantly across studies, ranging from 50 to 92.3%, highlighting the variability in participant engagement. Methodological quality was also variable, with allocation concealment and blinding being common limitations.

**Conclusion:**

Internet- and mobile-based interventions show promise for aftercare and relapse prevention in depression, with limited evidence for anxiety disorders. Future research should focus on optimizing engagement, personalizing interventions, standardizing outcome measures, and conducting larger trials with longer follow-up periods. These findings have important implications for integrating digital tools into existing care pathways to improve long-term outcomes for individuals with anxiety and depressive disorders.

**Systematic review registration:**

https://www.crd.york.ac.uk/prospero/display_record.php?ID=CRD42020151336, CRD42020151336.

## 1 Introduction

Depressive and anxiety disorders are common psychiatric conditions affecting psycho-social functioning and are characterized by complex and recurring clinical symptomatology. Major Depressive Disorder (MDD) features episodes lasting a minimum of 2 weeks, marked by notable mood, cognitive, and neurovegetative disruptions, affecting over 280 million individuals worldwide (Malhi and Mann, 2018). Anxiety disorders, with a global prevalence of 7.3%, are characterized by persistent fear and anxiety, associated with maladaptive avoidance behaviors and significant functional impairment (Craske and Stein, 2016). The substantial comorbidity between these disorders—approximately 60% of patients with MDD also presenting a comorbid anxiety disorder—is associated with increased symptom severity and unfavorable prognosis (Gold et al., 2020).

Generally, clinical evidence demonstrates that traditional pharmacological and psychological interventions are effective approaches for relapse prevention. Specifically, compliance with antidepressant treatments remains a cornerstone of relapse prevention, demonstrating efficacy in long-term follow-up studies (Bockting et al., 2015). Among psychological interventions, Cognitive Behavioral Therapy (CBT) and Mindfulness-Based Cognitive Therapy (MBCT) have emerged as effective approaches for preventing depressive and anxiety relapses (Kuyken et al., 2016).

Nonetheless, the critical need for effective aftercare is highlighted by substantial relapse rates in epidemiological data - up to 80% for MDD and between 24 and 58% for anxiety disorders within 2 years post-remission (Arias et al., 2022). Furthermore, long-term studies indicate that 8.5% of individuals with anxiety and depression remain chronic, and 32.9% experience intermittent relapses (Solis et al., 2021). Therefore, tertiary prevention, including aftercare and relapse prevention programs, are essential to sustain treatment gains and mitigate the negative impact of these disorders (Bockting et al., 2015, 2018; Huijbers et al., 2015).

Advances in digital technologies, including internet and mobile applications (IMIs), offer promising solutions to deliver scalable, accessible, and cost-effective aftercare interventions (Hennemann et al., 2018; Naslund et al., 2017). Emerging evidence suggests that these technology-enabled approaches can be effective in maintaining treatment gains and preventing relapse for anxiety and depressive disorders (Hennemann et al., 2018; Kuyken et al., 2016; Naslund et al., 2017). IMIs may offer advantages in terms of anonymity, flexibility, and the ability to provide personalized, interactive, and real-time support to individuals in the critical post-treatment period (Schlief et al., 2022). Previous research on IMIs for mental health has primarily focused on their use as primary treatment modalities (Ebert et al., 2018; Sander et al., 2016). However, a growing body of literature has begun to explore the potential for these technologies in aftercare and relapse prevention, with evidence suggesting they can effectively help patients sustain the benefits of treatment and prevent future relapses over the long term (Bockting et al., 2015, 2018; Hennemann et al., 2018; Huijbers et al., 2015; Kuyken et al., 2016).

To better understand the current state of the evidence on internet- and mobile-based aftercare and relapse prevention interventions for anxiety and depressive disorders, a comprehensive systematic review is warranted.

## 2 Method

### 2.1 Protocol and registration

This systematic review was conducted following the guidelines of the “Preferred Reporting Items for Systematic Reviews and Meta-Analyses” (PRISMA) (Page et al., 2021). The review protocol was registered and described in detail in the international prospective register of systematic reviews (PROSPERO: CRD42020151336).

### 2.2 Eligibility criteria

#### 2.2.1 Types of studies

We included randomized controlled trials (RCTs) and multi-arm studies that were available in full text. Non-randomized controlled and uncontrolled studies were excluded. RCTs were defined as trials that had an intervention and a control group, random assignment of participants, and specific measurable objectives and outcome measures. Studies published in English were included, and eligible publication types were journal articles, conference proceedings, conference abstracts, dissertations, and research reports. Completed but unpublished studies from trials registers were excluded.

#### 2.2.2 Types of participants

Studies focusing on adults (≥18 years) of any gender and ethnicity who have previously received acute treatment for anxiety and/or depressive disorders within the last 6 months were included. Eligible anxiety and depressive disorders included Major Depressive Disorder, Persistent Depressive Disorder, Premenstrual Dysphoric Disorder, Specific Phobia, Social Anxiety Disorder, Panic Disorder, Agoraphobia, and Generalized Anxiety Disorder. Studies with participants who had other comorbid mental disorders were included if anxiety/depressive disorders were among the main outcomes of the acute treatment. Studies with participants previously treated for a somatic condition with comorbid anxiety and/or depressive disorders were eligible if anxiety/depressive disorders were among the primary outcomes.

#### 2.2.3 Types of interventions

Interventions were defined as psychological programs provided predominantly in an online or mobile setting, including web pages, videoconference, chat, email, mobile applications, or text messages. The interventions were aimed at aftercare, follow-up treatment, maintenance, or relapse prevention for anxiety/depressive disorders. Interventions could vary in length, theoretical basis, and degree of human support (unguided/self-administered, minimal guidance, or online therapy). Interventions delivered solely through telephone calls or those which provided only psychoeducational content were excluded.

#### 2.2.4 Comparator interventions

Eligible comparators included inactive control groups (waiting list controls, no-treatment control, attention-placebo) and active control groups (face-to-face psychological intervention, telephone-delivered psychological intervention, pharmacological treatment, combined treatment, and other active treatments).

#### 2.2.5 Outcomes

Primary outcomes included the severity of anxiety and depressive symptoms, relapse of anxiety/depression, recurrence of anxiety/depression, and rehospitalization. Secondary outcomes included general quality of life and adherence to primary treatment.

### 2.3 Search strategy

We conducted systematic searches in the following databases: MEDLINE (via PubMed), CENTRAL (via The Cochrane Library), Scopus, Web of Science, PsycINFO, PsycARTICLES, PsycEXTRA, ProQuest Dissertations and Theses Open, Open Access Theses and Dissertations, and Open Grey. The initial search was conducted between April 6, 2019, and May 3, 2019, with a subsequent search performed between June 1, 2021, and June 4, 2021, to capture studies published after the initial search. The search strategy was designed according to the Population, Intervention, Comparison, Outcomes and Study (PICOS) framework and adapted for each database using a combination of subject headings and free text terms related to aftercare, internet- and mobile-based interventions, depressive/anxiety disorders, and randomized controlled trials (see Supplementary material S1 for the complete search strategy). To ensure the comprehensiveness of our review, we performed an additional check 10–15 December 2023. This check specifically focused on completed trials that were identified in our previous searches but had not been published at that time. We re-examined these previously identified completed trials to determine if they had been published since our last search. For each of these trials, we conducted targeted searches in scientific databases and through general web searches to locate any resulting publications. Any newly published studies from these previously identified completed trials were assessed for eligibility and, if relevant, included in our data extraction process.

### 2.4 Study selection

Retrieved records were managed using Mendeley. Two independent researchers [LP, MG] screened titles and abstracts to identify eligible studies. The researchers coded the records as eligible, ineligible, or unclear and resolved disagreements through discussion. Inter-rater reliability was calculated using the kappa statistic. Full texts of potentially eligible studies were then assessed for final inclusion.

### 2.5 Data extraction

Data were extracted using COVIDENCE. The following data were extracted from each study: (1) study identification, (2) study design characteristics, (3) intervention details, (4) primary diagnosis, (5) previous treatment type, (6) technical setting, (7) degree of professional support, (8) outcomes, (9) instruments, (10) results. Two independent researchers [LP, PP] extracted the data, and disagreements were resolved through discussion. Data from multiple reports of the same study were extracted into a single form.

### 2.6 Risk of bias assessment

The risk of bias was assessed using the Cochrane Collaboration’s tool (Higgins et al., 2011), covering the following domains: sequence generation, allocation concealment, blinding of participants, blinding of personnel, blinding of outcome assessment, incomplete outcome reporting, selective outcome reporting, other sources of bias. Each domain was rated as low, high, or unclear risk of bias. Disagreements were resolved through discussion.

### 2.7 Data synthesis

We performed a qualitative synthesis of the included studies. The synthesis focused on summarizing the characteristics and findings of the included studies and discussing the implications of the results. Heterogeneity among studies was assessed based on study design, participant characteristics, intervention details, and outcomes measured. Potential sources of heterogeneity were explored qualitatively.

## 3 Results

### 3.1 Study selection

**Figure 1:**
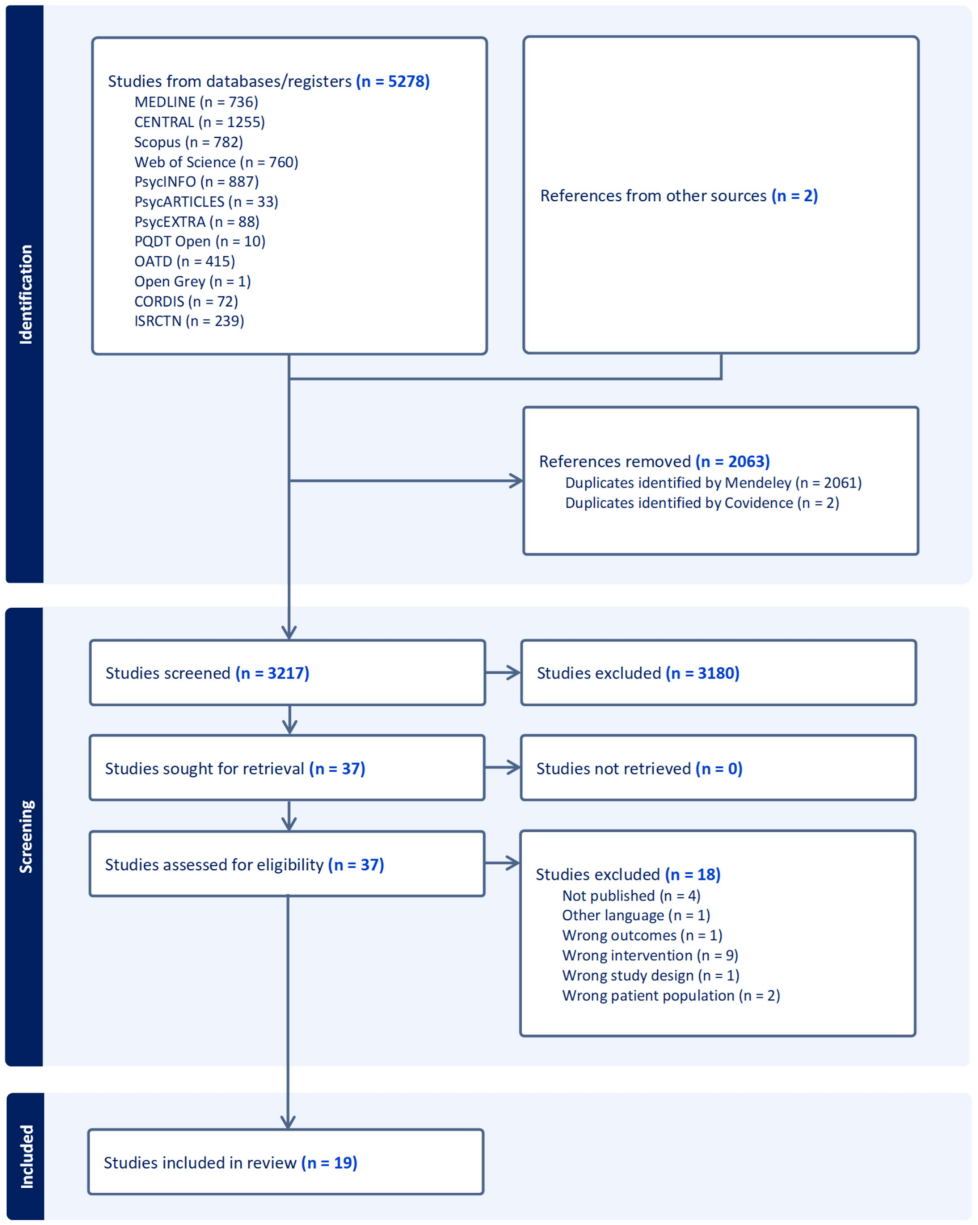
PRISMA flowchart of study inclusion process.

The systematic search of databases and registers yielded 5,278 studies, with an additional 2 references identified from other sources (Kok et al., 2014; Kraft et al., 2017). After removing 2,063 duplicates (2,061 identified by Mendeley and 2 by Covidence), 3,217 studies were retained for screening. Of these, 3,180 were excluded based on title and abstract review. The full texts of the remaining 37 studies were sought for retrieval, all of which were successfully obtained and assessed for eligibility. Following full-text review, 18 studies were excluded for the following reasons: not published (*n* = 4), other language (*n* = 1), wrong outcomes (*n* = 1), wrong intervention (*n* = 9), wrong study design (*n* = 1), and wrong patient population (*n* = 2). Ultimately, 19 studies met all inclusion criteria and were included in the review for qualitative synthesis. This selection process is illustrated in the PRISMA flow diagram (Figure 1).

### 3.2 Study characteristics

The systematic search yielded 19 randomized controlled trials (RCTs), published between 2011 and 2023, comprising a total of 3,206 participants. Sample sizes ranged from 41 (Kraft et al., 2017) to 460 participants (Segal et al., 2020), with a mean of 160.4 participants per study (SD = 123.7). The mean age of participants across studies ranged from 37.2 years (Browning et al., 2012) to 51.0 years (Vicent-Gil et al., 2019). Most studies included predominantly female samples, with the percentage of female participants ranging from 51.1% (O’Reilly et al., 2019) to 80% (Hunkeler et al., 2012).

Most studies (*n* = 16) primarily focused on major depressive disorder (MDD), while others included participants with anxiety disorders (*n* = 2), alcohol use disorder co-occurring with depression (*n* = 2), or persistent depressive disorder (n = 1). Eight studies specifically targeted participants in remission or with a history of depressive episodes. Study designs were predominantly parallel group randomized controlled trials (RCTs), with the number of arms ranging from two to five. Intervention durations varied widely from 2 weeks (Browning et al., 2012) to up to 12 months (Kordy et al., 2016), with follow-up periods extending to 24 months in some cases (Holländare et al., 2013). Studies were conducted in various settings, including outpatient, community, and post-inpatient contexts.

Most interventions (*n* = 16) were internet- or mobile-based, while five studies utilized primarily telephone-based interventions (e.g., van den Berg et al., 2015; Hunkeler et al., 2012). Professional support ranged from unguided self-help (e.g., Agyapong et al., 2012) to regular therapist contact (e.g., Vicent-Gil et al., 2022), with most studies (*n* = 12) providing some form of minimal guidance.

Interventions were diverse, including cognitive-behavioral therapy (CBT, e.g., Kok et al., 2015), mindfulness-based approaches (e.g., Kraft et al., 2017; Segal et al., 2020), attentional bias modification (Browning et al., 2012), and supportive text messaging (e.g., Agyapong et al., 2012; O’Reilly et al., 2019). Control conditions were predominantly treatment as usual (*n* = 12; e.g., Klein et al., 2018), with the remainder using wait-list controls (*n* = 4; e.g., Schlicker et al., 2017) or active comparators (*n* = 3; e.g., Browning et al., 2012).

Notably, the studies varied in their approach measuring relapse or recurrence. Some studies used structured clinical interviews (e.g., SCID; Klein et al., 2018), while others relied on self-report measures or predefined cut-off scores on depression scales (e.g., Holländare et al., 2013).

Table 1 presents a comprehensive summary of the characteristics of the studies included in this analysis.

**Table 1:** Summary of study characteristics.

Source	Type of RCT	Study setting	Diagnostic criteria	Inclusion criteria	Exclusion criteria
Vicent-Gil et al. (2022)	PG (3 arms)	Hospital	DSM-5	Age 18–60, history of MDD, currently in clinical remission or partial remission (HDRS <14), cognitive deficits (SCIP <80), psychosocial dysfunction (FAST ≥12)	IQ <85, medical conditions with neuropsychological impairment, comorbid psychiatric disorders, recent ECT or psychological intervention
Aggestrup et al. (2023)	PG (2 arms)	Outpatient	DSM-IV	Age ≥ 18 years, diagnosis of major depressive episode, MADRS-S score 15–35	Hamilton suicidal item 3 score ≥ 2, current substance abuse, comorbid dementia or brain disorders, bipolar disorder, psychotic depression
Agyapong et al. (2012)	PG (2arms)	Hospital	DSM-IV	Age > 18, MMSE score ≥ 25, DSM-IV criteria for major depressive disorder and alcohol dependence/abuse, completed inpatient dual diagnosis program	Bipolar disorder, psychotic disorder, current polysubstance dependence/abuse
Kraft et al. (2017)	PG (2 arms)	Hospital	ICD-10	Age 18–75, inpatient or day patient, depressive symptoms	Psychotic symptoms, history of schizophrenia, current mania, risk of dissociative crisis, severe cognitive impairment, severe substance abuse, suicidality, insufficient German language skills
van den Berg et al. (2015)	PG (3 arms)	Hospital	NS	Diagnosed depression, anxiety, adjustment or somatoform disorder; being discharged from day hospital	Interval patients, emotional instability with recurrent suicide crises/self-harm
Browning et al. (2012)	PG (2×2 design)	Outpatient	DSM-IV	Aged 18–65 years, ≥2 previous depressive episodes, currently in remission, MADRS-S score 15–35	Current major depression, suicidal ideation, current substance abuse, psychotic disorder
Kok et al. (2015)	PG (2 arms)	Recruited via media, practitioners	DSM-IV-TR	Age 18–65, ≥2 previous depressive episodes, in remission 2–24 months, HRSD score ≤ 10	Current depression, predominant anxiety disorder, bipolar disorder, psychosis, substance abuse
Kordy et al. (2016)	PG (multicenter)	Psychiatric departments	DSM-IV	Age 18–65, ≥3 depressive episodes, Internet access	Acute suicide risk, history of psychosis/bipolar disorder/organic brain disorder, primary diagnosis of another DSM-IV axis I disorder, severe medical conditions, severe cognitive impairment
Simon et al. (2011)	PG (2 arms)	Primary care clinics	NS	Age ≥ 18, new antidepressant prescription from primary care for depression, registered for online messaging	Antidepressant prescription in prior 270 days, bipolar/psychotic disorder diagnosis, mood stabilizer/antipsychotic prescription
Nyström et al. (2017)	PG (5 arms)	Internet-based	DSM-IV-TR	Age > 18, MADRS-S score 15–35, access to computer/internet, resident in Sweden, able to read/write Swedish	Severe depression, suicidal ideation, other primary psychiatric diagnosis, current psychological treatment, recent medication changes, active exercisers
Zwerenz et al. (2017)	PG (2 arms)	Inpatient clinic	NS	Inpatients/day clinic patients above 18 years with Internet access	Acute suicidality, psychosis, current substance addiction, lifetime diagnosis of schizophrenia, bipolar or organic psychiatric disorder
Klein et al. (2018)	PG (2 arms, single-blind)	Recruited via media, practitioners	DSM-IV	Age 18–65, ≥2 previous depressive episodes, in remission 8–24 months, HRSD score ≤ 10	Not specified
O’Reilly et al., 2019	PG	Outpatient	DSM-IV	Age 18–70, completed inpatient program, MMSE >25, BDI ≥14, in possession of mobile phone	Psychosis, primary substance abuse other than alcohol
Schlicker et al. (2017)	PG (3 arms)	Hospital	ICD-10	Age ≥ 18, MDD diagnosis, German fluency, basic reading/writing skills, mobile phone access	Psychotic diagnosis, acute substance dependence, significant suicide risk
Segal et al. (2020)	PG (3 arms)	Health clinics	PHQ-9, history of MDD	Age ≥ 18, history of major depressive disorder, current PHQ-9 score 5–9	Schizophrenia, bipolar disorder, current psychosis, organic mental disorder, pervasive developmental delay
Ebert et al. (2013)	PG (2 arms)	Post-inpatient, internet-based	ICD-10	≥18 years old, met criteria for a mental disorder according to ICD-10, fluent in German, basic reading and writing skills, access to internet	Psychotic diagnosis, acute alcohol or substance dependence, significant risk of suicide
Hunkeler et al. (2012)	PG (2 arms)	Mental health clinics	DSM-IV	Age ≥ 18, diagnosis of recurrent/chronic depression, not hospitalized, fluent in English, uses Internet at home	Bipolar disorder, current hospitalization
Holländare et al. (2013)	PG (2 arms)	Internet-based	DSM-IV	MADRS-S score 7–19, previous psychological or pharmacological treatment	Not explicitly stated
Hoorelbeke et al. (2015)	PG (2 arms)	Online/at-home	DSM-IV	History of depression, stable remission ≥6 months, age 18–65	Bipolar disorder, psychosis, substance abuse, brain injury, current comorbid disorders

	Sample size (N); Age (M, SD); % female	Primary diagnosis previous treatment type	Total study duration time points	Intervention details technical setting, degree of professional support duration
Vicent-Gil et al. (2022)	*N* = 52 51(7.14) 71.2%	MDD (remission) Pharmacological	9 months T0: Baseline T1: Post-intervention (3 months) T2: Follow-up (6 months after T1)	Cognitive and functional remediation (INCREM program), psychoeducation Internet-based, therapist-led 110-min sessions of functional remediation + computerized cognitive training (INCREM) or psychoeducation + non-directed game (psychoeducation) 12 weeks
Aggestrup et al. (2023)	*N* = 103 CRT:41.8(14.8), TAU:40.6 (14.8) 62%:51%	MDD Combined	4 weeks Baseline (at inclusion) Weekly phone calls End point (after 4 weeks)	Circadian Reinforcement Therapy (CRT) + electronic self-monitoring Combined, guided (psychoeducation sessions, daily electronic self-monitoring, weekly scheduled phone calls or meetings, plus additional calls triggered by predefined alert points in the MDB system) 4 weeks
Agyapong et al. (2012)	*N* = 54 IG:48(10.4), CG: 49.1(10.5) 53.7%	MDD and AUD Combined	3 months Baseline (at discharge) 3-month follow-up	Supportive text messages Mobile-based, unguided 180 supportive text messages targeting mood, medication adherence, and alcohol abstinence 4 months
Kraft et al. (2017)	*N* = 41 IG:43.4(12.7), CG: 44.5 (13.5) 68.3%	MDD; PDD NS	4 months Baseline (pre-randomization) Prerandomization (before discharge) 4-month follow-up	Mindfulness training & text message support Mobile-based, guided (automated text message feedback) Manualized group introduction to 3 mindfulness exercises during inpatient treatment, followed by daily home practice for 4 months. Intervention group received reinforcing text message feedback. 17 weeks
van den Berg et al. (2015)	*N* = 123 44 (12.5) 71.5%	MD/ND-SRD Psychological	6 months Baseline (at discharge from day hospital) Follow-up (6 months after baseline) and 26 weeks	Telephone contacts plus text messages Mobile-based, guided (calls conducted by trained nurses) Weekly calls in first month, then monthly calls for 5 months; weekly individualized text messages (group 2 only) 26 weeks
Browning et al. (2012)	*N* = 61 IG_1_:34.6 (12.2), IG_2_:40.9 (11.3), IG_3_:37.8 (11.5), IG_4_:40.9 (13.5) 65.6%	MDD-R No psychotropic or psychological treatment (last 2 months)	6 weeks Baseline, post-intervention (2 weeks), 4-week follow-up	Cognitive bias modification: Positive face-based ABM & Positive word-based ABM vs. Neutral face-based ABM & Neutral word-based ABM Computer-based, unguided Computerized attentional bias modification tasks completed twice daily 2 weeks
Kok et al. (2015)	*N* = 239 CT: 45.52(10.9), TAU:47.48(10.8) 79.4%:69.9%	MDD Combined	3 months Baseline, 1.5, and 3 months	CBT, mobile cognitive therapy (mobile CT) Internet-based, Minimal therapist support via telephone and email 8 online modules based on preventive cognitive therapy, 20 min per module plus homework 8 weeks
Kordy et al. (2016)	*N* = 232 NS 78%	MDD Combined	24 months Baseline (hospital discharge), 6, 12, 18, and 24 months	Internet-delivered disease management: SUMMIT (Internet-delivered augmentation), SUMMIT-PERSON (Internet-delivered augmentation with personal guidance) Internet-based, fully automated (SUMMIT) or with option of expert chats (SUMMIT-PERSON) Supportive monitoring, crisis management plan, Internet discussion forum 52 weeks
Simon et al. (2011)	*N* = 208 IG:49(13), CG:45(14) 69%:75%	MDD Pharmacological	6 months Baseline, 5 months. Intervention contact: 2, 6, 10 weeks	Collaborative care management program Internet-based, therapist-led Three online care management contacts at 2, 6 and 10 weeks including structured assessment, algorithm-based feedback, and facilitation of follow-up care 10 weeks
Nyström et al. (2017)	*N* = 286 42(13.5) 76%	MDD Psychological	12 weeks Baseline, weekly, post-treatment (12 weeks)	Behavioral activation Lewinsohn’s model (BAL), Martell’s model (BAM), physical activity (PA) with/without rationale Internet-based, therapist-led 12-week program, 8 modules, weekly therapist support via email
Zwerenz et al. (2017)	*N* = 69 43.06(12.36) 71%	MDD Psychological	20 weeks Baseline (at discharge from inpatient/day clinic treatment); 10 weeks (end of intervention for IG, end of waiting period for WL); 18 weeks (2-month follow-up, IG only); 20 weeks (end of intervention for WL)	Psychodynamic intervention Internet-based, feedback from trained psychologist 8 units over 10 weeks, based on affect phobia therapy model
Klein et al. (2018)	*N* = 264 45(11.5) 66%	MDD-R Pharmacological	24 months Baseline, 3, 12, and 24 months; 10 intervals for depressive symptoms	CBT: Mobile cognitive therapy (M-CT) Internet-based, minimal quidance (2 telephone sessions with psychologist), 8 online weekly modules based on preventive cognitive therapy, 20 min per module plus homework 8 weeks
O’Reilly et al., 2019	*N* = 95 48 (10.5) 53.7%	MDD NS	12 months Baseline; 3 months; 6 months (end of intervention); 12 months (6-month follow-up)	Supportive text messages focused on mood and alcohol abstinence sent twice daily for 6 months. Mobile-based, unguided 26 weeks
Schlicker et al. (2017)	*N* = 226 IG_1_:42.42(8.88), IG_2_:44.59(9.80) CG:44.60(10.97) 64.1%	MDD Combined	10 weeks Baseline (discharge from inpatient treatment); 6 weeks (post-intervention);10 weeks (follow-up)	CBT, standardized text messages focused on emotion regulation (TMMI-Dsta), individualized text messages (TMMI-Dind) Mobile-based, unguided, 1–5 text messages per day 6 weeks
Segal et al. (2020)	*N* = 460 48.3(14.9) 75.6%	MDD Combined	6 months Baseline, 1, 2, 3, and 6 months	Mindfulness-based intervention: Mindful Mood Balance (MMB) online program + usual depression care 8 online sessions of MMB + minimal phone/email coaching support
Ebert et al. (2013)	*N* = 400 IG:45(8.88) CG:45(9.80) 74.5%	F3, F4, F5 (ICD-10) Combined	16 months Baseline, post-treatment (3 months), 12-month follow-up	Transdiagnostic Internet-based Maintenance Treatment (TIMT) Internet-based, guided (weekly asynchronous written online feedback from a therapist) 12-week TIMT program including goal setting, action planning, peer support, coaching, and symptom monitoring
Hunkeler et al. (2012)	*N* = 103 IG:48.49(12.83) CG:51.88(10.56) 79.5%	MDD; PDD Combined	24 months Baseline, 6, 12, 18, and 24 months	Care management and cognitive-behavioral therapy strategies: eCare for Moods (Web-based care management + usual care) & usual specialty mental health care 12-month access to secure website with self-monitoring, messaging, education modules, discussion group
Holländare et al. (2013)	*N* = 84 45.3(12.8) 84.5%	MDD; PRUD Combined	24 months Baseline, post-treatment (10 weeks), 6-, 12-, and 24-month follow-up.	Internet-based CBT (iCBT) Internet-based, guided (personal therapist via encrypted emails) 16 CBT-based modules (9 mandatory, 7 optional) administered via secure internet platform 10 weeks
Hoorelbeke et al. (2015)	*N* = 68 IG:46.12(10.80) CG:47.82(12.20) 66.18%	MDD Combined	3 months Baseline, post-intervention (2 weeks), 3-month follow-up	Cognitive control training (adaptive PASAT) Internet-based, unguided 10 online sessions, 400 trials per session 2 weeks

AUD, Alcohol Use Disorder; BDI, Beck Depression Inventory; CBT, Cognitive-behavioral Therapy; CG, control group; CT, Cognitive Therapy; DSM, Diagnostic and Statistical Manual of Mental Disorders; FAST, Functioning Assessment Short Test; HDRS, Hamilton Depression Rating Scale; ICD, International Classification of Diseases; IG, intervention group; M, mean; MADRS-S, Montgomery-Asberg Depression Rating Scale; MD, Mood Disorders; MDD, Major Depressive Disorder; MDD-R, Major Depressive Disorder- Recurrent; MMSE, Mini-Mental State Examination; ND-SRD, Neurotic/Stress-Related Disorders; NS, not specified; PDD, Persistent Depressive Disorder; PG, parallel group; PHQ-9, Patient Health Quotient-9; PRUD, Partially Remitted Unipolar Depression; SD, standard deviation; TAU, treatment as usual; WL, waiting list.

Primary outcomes measured depressive symptoms, most assessed using the Beck Depression Inventory (BDI), Patient Health Questionnaire (PHQ-9) or Hamilton Depression Rating Scale (HDRS). Secondary outcomes often included anxiety symptoms, quality of life measures, and relapse rates (see Table 2). Participant attrition was reported in 17 of the 19 included studies, with varying levels of detail provided.

**Table 2:** Summary of study results.

	Instruments	Results
Vicent-Gil et al. (2022)	FAST SCIP PDQ-20 HDRS-17 RDQ SF-36	Final number of participants: INCREM: 9; Psychoeducation: 9; TAU: 8 Dropout rate: Overall: 50% (26 out of 52 completed); Per group rates not provided FAST (psychosocial functioning): Baseline values (mean ± SD): INCREM: 21.22 ± 4.12, Psychoeducation: 28.78 ± 8.84, TAU: 24.5 ± 6.09; Follow-up values (mean ± SD): INCREM: 9.33 ± 7.35, Psychoeducation: 24.11 ± 15.53, TAU: 21.63 ± 10.68; Between-group difference: Not directly reported; *p*-value: Significant difference between INCREM and psychoeducation at follow-up (*p* = 0.041) SCIP (cognitive performance): Baseline values (mean ± SD): INCREM: 66.78 ± 10.34, Psychoeducation: 74 ± 3.67, TAU: 64.5 ± 9.02; Follow-up values (mean ± SD): INCREM: 76.78 ± 9.38, Psychoeducation: 79.56 ± 7.80, TAU: 70.38 ± 11.41; Between-group difference: Not directly reported; p-value: Significant univariate treatment effect (*F* = 3.97; df = 2, 23; *p* = 0.03) Adverse effects: Not reported. Effect size: Not reported.
Aggestrup et al. (2023)	HAM-D17 MDI WHO-5 MEQ PSQI SUS MDB Fitbit tracker	Final number of participants: CRT group: 40 (for primary analyses); TAU group: 46 (for primary analyses) Dropout rate: CRT group: 9 out of 49 (18.4%); TAU group: 6 out of 52 (11.5%) HAM-D17 scores: Baseline values: CRT: 15.8 ± 0.9; TAU: 15.5 ± 0.8; Follow-up values (estimated): CRT: 12.8 ± 0.7; TAU: 13.6 ± 0.6; Between-group difference: 2.6 points (95% CI not provided); p-value: 0.04 MDI scores: Baseline values (whole sample mean): 21.5 ± 1.2; Follow-up values (estimated): CRT: 18.4 ± 1.1; TAU: 20.6 ± 1.1; Between-group difference: Not explicitly stated; p-value: Not significant (exact value not provided) Evening mood: *p* = 0.02; Sleep quality: p = 0.04; Sleep onset: 26.6 min earlier in CRT group, *p* = 0.009; Sleep duration: 0.48 h longer in CRT group, *p* = 0.005 Adverse effects: Two non-fatal overdose incidents due to suicidal ideation, both in the TAU group. Effect size: not explicitly reported.
Agyapong et al. (2012)	BDI-II TLFB GAF OCDS AASES	Final number of participants: IG = 24, CG = 26 Dropout rate: IG = 2/26 (7.7%); CG: 2/28 (7.1%) BDI-II scores at 3 months: Baseline values: IG: 31.58 ± 7.7; CG: 31.99 ± 9.5; Follow-up values: IG: 8.6 ± 7.9; CG: 16.6 ± 9.8; Between-group difference: −7.9 (95% CI: −13.06 to −2.76); *p* = 0.003 Cumulative Abstinence Duration (CAD) in days at 3 months: Follow-up values: IG: 88.3 ± 6.2; CG: 79.3 ± 24.1; *p* = 0.08 GAF scores at 3 months: Baseline: IG: 48.2 ± 4.9, CG: 48.6 ± 8.1; Follow-up: IG: 89.8 ± 12.2, CG: 76.1 ± 15.3; Between-group difference: 13.69 (95% CI: 5.71 to 21.66); *p* = 0.001 OCDS scores at 3 months: Baseline: IG: 26.0 ± 6.5; CG: 23.7 ± 6.0; Follow-up: IG: 8.4 ± 6.4; CG: 6.8 ± 4.4; Between-group difference: −1.41 (95% CI: −4.65 to 1.84); *p* = 0.40 AASES scores at 3 months: Baseline: IG: 38.9 ± 13.8, CG: 43.9 ± 9.8; Follow-up: IG: 79.5 ± 15.9, CG: 72.3 ± 14.7; Between-group difference: 7.84 (95% CI: −1.15 to 16.84); *p* = 0.09 Adverse effects: Not reported; Effect sizes: BDI-II: Cohen’s *d* = 0.85, CAD: Cohen’s *d* = 0.51, GAF: Cohen’s *d* = 1.02, OCDS: Cohen’s *d* = 0.18, AASES: Cohen’s *d* = 0.79.
Kraft et al. (2017)	PHQ-9 PTQ FMI SCS-D	Final number of participants: IG: 18, CG: 17, Total: 35 Dropout rate: IG: 14% (3/21), CG: 15% (3/20) Number of mindfulness exercises practiced PHQ-9 (depressive symptoms): Baseline values (mean ± SD): IG: 12.74 ± 5.69, CG: 18.61 ± 4.86; Follow-up values (mean ± SD): IG: 8.94 ± 6.61, CG: 12.06 ± 7.24; Between-group difference: Not reported; p-value: 0.68 Adverse effects: Not reported Effect sizes: Number of exercises practiced: *d* = 0.25 (95% CI: −0.45 to 0.96), PHQ-9: *d* = 0.14 (95% CI: −0.53 to 0.82), PTQ: *d* = −0.26 (95% CI: −0.84 to 0.31), FMI: *d* = 0.25 (95% CI: −0.49 to 0.99), SCS-D: *d* = 0.02 (95% CI: −0.59 to 0.63)
van den Berg et al. (2015)	BSI-18	Final number of participants: IG_1_: 41; IG_2_: 37; CG: 35; Total: 113 Dropout rate: IG_1_:1/42 (2.4%); IG_2_: 3/40 (7.5%); CG: 6/41 (14.6%) Anxiety: Baseline (mean ± SD): IG_1_: 7.60 ± 5.09, IG_2_: 7.43 ± 3.71, CG: 5.71 ± 4.79; Follow-up (mean ± SD): IG_1_: 6.71 ± 5.69, IG_2_: 5.38 ± 4.02, CG: 6.37 ± 5.80; Between-group differences: IG_1_vs CG: −0.87 (95% CI: −2.76 to 1.01), *p* = 0.364, IG_2_ vs. CG: −2.04 (95% CI: −3.99 to −0.076), *p* = 0.042 Depression: Baseline (mean ± SD): IG_1_: 8.71 ± 5.22, IG_2_: 7.10 ± 5.18, CG: 5.61 ± 5.26; Follow-up (mean ± SD), IG_1_: 6.27 ± 5.75, IG_2_: 6.22 ± 5.59, CG: 6.06 ± 5.70; Between-group differences: IG_1_ vs. CG: −1.73 (95% CI: −3.78 to 0.31), *p* = 0.097, IG_2_ vs. CG: −0.87 (95% CI: −2.90 to 1.17), *p* = 0.403 Somatization: Baseline (mean ± SD): IG_1_: 4.73 ± 4.07, IG_2_: 5.23 ± 3.77, CG: 3.90 ± 4.59; Follow-up (mean ± SD): IG_1_: 4.76 ± 3.90, IG_2_: 4.70 ± 3.99, CG: 3.91 ± 4.31; Between-group differences: IG_1_vs CG: 0.49 (95% CI: −1.05 to 2.04), *p* = 0.536, IG_2_ vs. CG: 0.17 (95% CI: −1.45 to 1.78), *p* = 0.838 Adverse effects: Not reported. Effect size: Not directly reported but can be calculated from the between-group differences and confidence intervals provided.
Browning et al. (2012)	BDI HRSD CAR STAI Visual probe task	Final number of participants: Positive face ABM: 16, Placebo face ABM: 14, Positive word ABM: 16, Placebo word ABM: 15 Dropout rate: 1 participant (group not specified) did not attend the final assessment session BDI score: Baseline values (mean ± SD): Positive face ABM: 5.9 ± 6.9, Placebo face ABM: 4.3 ± 3.7, Positive word ABM: 6.3 ± 5.4, Placebo word ABM: 3.8 ± 4.0; Follow-up values: Not directly reported, but graphs show: Positive face ABM: Decreased by ~2 points, Placebo face ABM: No significant change, Word ABM groups: No significant changes; Between-group difference: Not reported; *p*-value: p = 0.03 for face-based ABM effect HRSD score: Similar pattern to BDI but only trend-level significance (p = 0.09); STAI score: Significant reduction for positive face ABM group (p = 0.03); CAR: Significant reduction for positive face ABM vs. placebo (p = 0.03); Attentional bias: Significant increase in positive bias for positive face ABM group (*p* = 0.002) Adverse effects: Not reported. Effect sizes: Not directly reported Positive face-based ABM reduced depressive symptoms (BDI), anxiety symptoms (STAI), and cortisol awakening response compared to placebo, with effects emerging during the 1-month follow-up period. Word-based ABM did not show significant effects.
Kok et al. (2015)	IDS-SR30 HRSD-17 SCID-I	Final number of participants: Mobile CT: 126, TAU: 113 Dropout rate: Mobile CT: 16/126 (12.7%), TAU: 18/113 (15.9%) IDS-SR30 score: Baseline values (mean ± SD): Mobile CT: 16.44 ± 10.5, TAU: 16.06 ± 9.5; 3-month follow-up values (mean ± SD): Mobile CT: 16.38 ± 10.9, TAU: 21.52 ± 12.4; Between-group difference: 1.60 points per month (95% CI: −2.64 to −0.56); *p*-value: 0.003 Adverse effects: Not reported. Effect size: Cohen’s *d* = 0.44 (ITT analysis), Cohen’s *d* = 0.54 (Completer analysis) Mobile CT resulted in a small but statistically significant decrease in depressive symptoms over 3 months compared to TAU alone in remitted recurrently depressed patients.
Kordy et al. (2016)	SCID LIFE PSR PHQ-9	Final number of participants: TAU: 78, SUMMIT: 75, SUMMIT-PERSON: 79 Dropout rate: TAU: 2.5% (2/80), SUMMIT: 2.6% (2/77), SUMMIT-PERSON: 0% (0/79) “Well weeks” (weeks with PSR ≤2) vs. “unwell weeks” (PSR ≥3) over 24 months: Baseline values: Not reported; Follow-up values (median % of well weeks): TAU: 31%, SUMMIT: 52%, SUMMIT-PERSON: 48%; Between-group differences: SUMMIT vs. TAU: OR 0.48 (95% CI 0.23–0.98), SUMMIT-PERSON vs. TAU: OR 0.62 (95% CI 0.31–1.24), SUMMIT-PERSON vs. SUMMIT: OR 0.77 (95% CI 0.38–1.56), *p*-values: SUMMIT vs. TAU: p = 0.04, SUMMIT-PERSON vs. TAU: *p* = 0.18, SUMMIT-PERSON vs. SUMMIT: *p* = 0.47 Adverse effects: 169 serious adverse events reported (mostly rehospitalizations), equally distributed across groups. 3 suicide attempts/self-injuries (2 in SUMMIT, 1 in TAU). Effect size: Not explicitly reported, but odds ratios provided for primary outcome.
Simon et al. (2011)	SCL-20 PHQ-9 Single-item satisfaction rating	Final number of participants: Care management group (CM): 104, Usual care group (TAU): 93 Dropout rate: CM: 2/106 = 1.9%, TAU: 9/102 = 8.8% SCL depression score at 5 months: Baseline values not reported; Follow-up values: CM: 0.95 ± 0.71, TAU: 1.17 ± 0.81; Adjusted difference: 0.29 (95% CI: 0.06 to 0.51); *p* = 0.043 Satisfaction with depression treatment (% “very satisfied”): Baseline values not reported; Follow-up values: CM: 53% (56/104), TAU: 33% (31/93); Not reported; *p* = 0.004 Antidepressant adherence (% using antidepressant for >90 days): Baseline values not applicable; Follow-up values: CM: 81% (86/106), TAU: 61% (62/102); Not reported; p = 0.001 Adverse effects: No harms or unintended effects reported. No psychiatric hospitalizations or suicide attempts in either group. Effect size: 0.29 for SCL score
Nyström et al. (2017)	MADRS-S PHQ-9 GAD-7 IPAQ QOLI SCID-I	Final number of participants: 286 total Dropout rate: Overall dropout rate: 8.3% (26/312); dropout rates per group were not reported; the study used an intention-to-treat analysis, including all participants who provided data for at least one weekly measure or the post-treatment evaluation (286 participants) PHQ-9 scores. Baseline values (mean ± SD): All treatment groups combined: 12.81 ± 4.58, CG: 12.01 ± 5.08; Follow-up values at 12 weeks (mean ± SD): All treatment groups combined: 7.61 ± 6.17, CG: 9.26 ± 6.45; Between-group difference: When comparing all treatment groups combined to control, treatment predicted a steeper decline in depression scores (*B* = −0.669, SE = 0.198, *p* = 0.001, 95% CI [−1.058, −0.281]); *p*-value: 0.001 GAD-7 scores. Baseline values (mean ± SD): All treatment groups combined: 9.28 ± 4.63, CG: 8.79 ± 4.62; Follow-up values at 12 weeks (mean ± SD): All treatment groups combined: 5.64 ± 4.12, CG: 6.61 ± 5.31; Between-group difference: Treatment predicted a steeper decline in anxiety scores compared to control (*B* = −0.384, SE = 0.169, *p* = 0.023, 95% CI [−0.716, −0.052]); p-value: 0.023 Adverse effects: Not reported. Effect sizes (Hedges gav): PHQ-9: All treatments combined: 1.01, GC: 0.47; GAD-7: All treatments combined: 0.83, GC: 0.43
Zwerenz et al. (2017)	CSQ-8 ERSQ PHQ-9 GAD-7 CDS-2 EUROHIS-QOL-8 RSE SSS-8 SPE	Final number of participants: Intervention group (IG): 36, Wait-list control group (WL): 33 Dropout rate: IG: 4/42 (9.5%), WL: 3/40 (7.5%) Satisfaction with the intervention (CSQ-8 item): 95% rated as “very satisfied” or “mostly satisfied” Depression (PHQ-9): IG: 11.92 ± 5.46, WL: 12.06 ± 5.7; IG: 11.06 ± 6.49, WL: 13.15 ± 5.89; Not reported; p = 0.02 Quality of life (EUROHIS-QOL-8): IG: 2.04 ± 0.69, WL: 1.98 ± 0.62; IG: 2.15 ± 0.88, WL: 1.87 ± 0.66; Not reported; p = 0.04 Emotional competence (ERSQ): IG: 61.75 ± 17.13, WL: 60.09 ± 15.22; IG: 63.84 ± 18.24, WL: 56.24 ± 15.60; Not reported; *p* = 0.05 (trend) Adverse effects were not reported. Effect sizes (Cohen’s d): Depression: *d* = 0.60; Quality of life: *d* = 0.53; Emotional competence: *d* = 0.49
Klein et al. (2018)	SCID-I HRSD IDS-SR	Final number of participants: Mobile Cognitive Therapy (M-CT) + Treatment as Usual (TAU): 132 - TAU alone: 132 Dropout rate: 29 participants dropped out immediately after randomization; 24 were lost to follow-up (Specific dropout rates per group not provided) Time to relapse/recurrence according to DSM-IV criteria assessed with SCID-I: Baseline values: Not reported; Follow-up values (24 months): Cumulative relapse/recurrence rate: M-CT: 44%, TAU: 49%; Between-group difference: Hazard ratio = 0.77, 95% CI = 0.53–1.14; p-value: 0.190 Number of relapses/recurrences: Incidence rate ratio = 0.87, 95% CI = 0.64–1.19, *p* = 0.393 Depressive symptoms (IDS-SR): B = 0.31, 95% CI = −0.09-0.70, *p* = 0.131 Adverse effects: Not reported. Effect size: Not reported but can be inferred from the hazard ratio of 0.77 for the primary outcome, indicating a small effect size M-CT that was not statistically significant.
O’Reilly et al., 2019	MMSE SCID TLFB BDI-II BAI PSS OCDS	Final number of participants: IG: 47, CG: 48 Dropout rate: IG: 25.5%, CG: 43.7% Change in units of alcohol per drinking day. Baseline values (mean ± SD): IG: 16.5 ± 7.7, CG: 14.1 ± 5.0; 6-month follow-up (mean ± SD): IG:12.4 ± 8.6, CG: 8.0 ± 7.9; Between-group difference not reported; *p* = 0.03 BDI-II scores. Baseline values (mean ± SD): IG: 31.0 ± 12.0, CG: 29.9 ± 10.8; 3-month follow-up (mean ± SD): IG: 19.8 ± 12.3, CG: 13.0 ± 15.1; Between-group difference not reported; *p* = 0.02 PSS score. Baseline values (mean ± SD): IG: 27.6 ± 6.3, CG: 26.6 ± 6.5; 3-month follow-up: Significant interaction effect between group and time *F* (1,85) = 3.9, *p* = 0.05 Adverse effects: Not reported. Effect sizes: Units per drinking day at 6 months: *r* = 0.3 (medium effect); BDI-II at 3 months: *r* = 0.3 (medium effect); PSS at 3 months: partial η^2^ = 0.04 (small effect).
Schlicker et al. (2017)	BDI-II PANAS GSES ERSQ	Final number of participants: TMMI-Dsta (standardized text messages): 77, TMMI-Dind (individualized text messages): 73, WL: 76 Dropout rates: TMMI-Dsta: 30.26% at follow-up, TMMI-Dind: 36.98% at follow-up, WL: 36.36% at follow-up BDI-II scores. Baseline values (mean ± SD): TMMI-Dsta: 16.02 ± 11.80, TMMI-Dind: 14.13 ± 11.09, WL: 15.59 ± 12.09; Follow-up values (mean ± SD): TMMI-Dsta: 13.06 ± 10.18, TMMI-Dind: 16.30 ± 11.63, WL: 18.29 ± 13.93; Between-group differences (95% CI): TMMI-Dsta vs. WL: 4.23 (0.80 to 7.66), TMMI-Dind vs. WL: 0.12 (−3.62 to 3.86), TMMI-Dsta vs. TMMI-Dind: 4.28 (1.06 to 7.49); p-values not explicitly reported, but TMMI-Dsta vs. WL difference was significant Negative Affect (PANAS): Baseline: TMMI-Dsta 1.32 ± 0.83, TMMI-Dind 1.20 ± 0.69, WLC 1.27 ± 0.86; Follow-up: TMMI-Dsta 1.16 ± 0.76, TMMI-Dind 1.20 ± 0.80, WL 1.22 ± 0.88; TMMI-Dsta vs. WL: 0.09 (−0.14 to 0.34), TMMI-Dind vs. WLC: −0.01 (−0.29 to 0.26); No significant differences reported Positive Affect, Self-Efficacy, and Emotion Regulation Skills showed no significant between-group differences. Adverse effects: Not reported. Effect sizes: BDI-II: TMMI-Dsta vs. WL: *d* = 0.44 at follow-up, TMMI-Dind vs. WL: *d* = 0.17 at follow-up, TMMI-Dsta vs. TMMI-Dind: *d* = 0.28 at follow-up.
Segal et al. (2020)	PHQ-9 GAD-7 SF-12	Final number of participants: Mindful Mood Balance (MMB) + Usual Depression Care (UDC): 154, UDC only: 176 Dropout rate: MMB + UDC: 33.0% (76/230), UDC only: 23.5% (54/230) PHQ-9 scores. Baseline values (mean ± SD): MMB + UDC: 7.20 ± 1.4, UDC only: 7.29 ± 1.53; 15-month follow-up values (mean ± SD): MMB + UDC: 5.10 ± 4.19, UDC only: 7.06 ± 4.76; Between-group difference: 0.95 (SE 0.39); *p*-value: *p* < 0.02 Anxiety symptoms (GAD-7). Baseline values (mean ± SD): MMB + UDC: 6.51 ± 3.15, UDC only: 6.20 ± 3.28; 15-month follow-up values (mean ± SD): MMB + UDC: 3.49 ± 3.21, UDC only: 4.92 ± 4.23; Between-group difference: 1.21 (SE 0.42); *p*-value: *p* = 0.004 Mental functioning (SF-12 MCS). Baseline values (mean ± SD): MMB + UDC: 34.27 ± 7.92, UDC only: 34.22 ± 8.63; 15-month follow-up values (mean ± SD): MMB + UDC: 44.37 ± 10.51, UDC only: 39.64 ± 11.93; Between-group difference: −5.10 (SE 1.37); *p*-value: *p* < 0.001 Adverse effects: 1 serious adverse event (overdose) reported in MMB + UDC group, none in UDC only group. Effect size: For PHQ-9 scores over entire study period: Cohen’s *d* = 0.23 (95% CI: 0.04 to 0.41)
Ebert et al. (2013)	HEALTH-49 PANAS ERSQ	Final number of participants: TIMT + TAU: 131 at 12-month follow-up, TAU-only: 146 at 12-month follow-up Dropout rate: TIMT + TAU: 34.5% at 12-month follow-up, TAU: 27% at 12-month follow-up General psychopathological symptom severity (HEALTH-49 GPS subscale). Baseline values (mean ± SD): TIMT + TAU: 0.81 ± 0.70, TAU: 0.82 ± 0.70; 12-month follow-up values (estimated from model): TIMT + TAU: 0.76, TAU: 1.13; Between-group difference at 12 months: −0.36 (95% CI: −0.50 to −0.22); *p*-value: *p* < 0.001 Depression (HEALTH-49 Depression subscale). Baseline values (mean ± SD): TIMT + TAU: 0.91 ± 0.80, TAU: 0.97 ± 0.81; 12-month follow-up values (estimated from model): TIMT + TAU: 0.96, TAU: 1.38; Between-group difference at 12 months: −0.36 (95% CI: −0.56 to −0.15); *p*-value: *p* < 0.001 Adverse effects: Not explicitly reported. Effect sizes: GPS at 12 months: *d* = 0.55; Depression at 12 months: *d* = 0.33 to 0.56
Hunkeler et al. (2012)	PSR SCID LIFE Sheehan DS SF-36 AUDIT Custom satisfaction scales	Final number of participants: eCare group: 49, Usual care group (TAU): 51 Dropout rate: eCare: 2/51 (3.9%), TAU: 1/52 (1.9%) Depression severity over 2 years (PSR scale). Baseline (mean ± SD): eCare: 3.88 ± 1.21, TAU: 3.65 ± 1.18; Follow-up at 24 months (mean ± SD): eCare: 2.95 ± 1.11, TAU: 3.11 ± 1.08; Between-group difference: −0.74 (95% CI: −1.38 to −0.09); *p*-value: 0.025 Depression presence (PSR ≥3): Between-group difference: −0.24 (95% CI: −0.46 to −0.03), *p*-value: 0.026 SF-36 Mental Health: Baseline (mean ± SD): eCare: 36.65 ± 12.43, TAU: 40.51 ± 9.29; 24 months (mean ± SD): eCare: 41.91 ± 13.24, TAU: 40.58 ± 9.70, *p*-value: 0.002 Adverse effects: Not specifically reported. Effect size: Cohen’s *d* = 0.60 depression severity
Holländare et al. (2013)	SCID-I MADRS-S BDI-II BAI WHOQOL-BREF	Final number of participants: iCBT: 32, CG: 35 Dropout rate: iCBT: 23.8% (10/42), CG: 16.7% (7/42) Relapse rates. Baseline: N/A; 24-month follow-up: iCBT: 13.7% (95% CI 2.5–24.9%), CG: 60.9% (95% CI 44.8–77%); Between-group difference not directly reported; *p* < 0.001 Symptom levels MADRS-S. Baseline: iCBT: 13.7 ± 5.8, CG: 15.0 ± 5.8; 24-month follow-up: iCBT: 7.4 ± 6.4, CG: 8.9 ± 6.6; Between-group effect size: *d* = 0.03 (95% CI -0.40 to 0.46); *p* = 0.059 for group x time interaction Adverse effects: Not reported. Effect size: MADRS-S: *d* = 0.03 (95% CI -0.40 to 0.46), BDI-II: *d* = 0.36 (95% CI -0.07 to 0.79)
Hoorelbeke et al. (2015)	PASAT BRIEF-A RRS BDI-II CERQ RS QLDS WHODAS 2.0 RDQ	Final number of participants: Cognitive control training (CCT) group: *n* = 34, Active control group (AC): *n* = 34 Dropout rate: Not explicitly reported Brooding (RRS subscale) and depressive symptomatology (BDI-II). Baseline values (mean ± SD): Brooding. Baseline values (mean ± SD): CCT: 10.29 ± 3.77, AC: 10.35 ± 2.91; Follow-up at 3 months (mean ± SD): CCT: 7.12 ± 1.95, AC: 9.44 ± 3.23; Between-group differences at follow-up: 2.32 (95% CI: 1.03, 3.62); *p* = 0.001 Depressive symptoms: CT: 8.77 ± 8.65, AC 7.27 ± 6.28; CCT: 4.50 ± 5.10, AC: 9.29 ± 7.28; 4.79 (95% CI: 1.75, 7.84); *p* = 0.002 Maladaptive emotion regulation (CERQ): Baseline: CT: 36.21 ± 9.41, AC: 36.24 ± 10.86; Follow-up: CCT: 26.56 ± 7.88, AC: 32.91 ± 9.47; Between-group difference: 6.35 (95% CI: 2.14, 10.57); *p* = 0.004 Resilience (RS): Baseline: CCT: 76.41 ± 10.37, AC: 75.50 ± 11.32; Follow-up: CCT: 82.94 ± 11.98, AC: 75.53 ± 11.52; Between-group difference: 7.41 (95% CI: 1.72, 13.10); *p* = 0.011; Adverse effects: Not reported. Effect sizes: Brooding: *d* = 0.87, Depressive symptoms: *d* = 0.76, Maladaptive emotion regulation: *d* = 0.73, Resilience: *d* = 0.63

AASES, Alcohol Abstinence Self-Efficacy Scale; ABM, Attentional Bias Modification; BDI-II, Beck’s Depression Inventory; BSI-18, Brief Symptom Inventory-18; CAR, Cortisol Awakening Response; CERQ, Cognitive Emotion Regulation Questionnaire; CG, control group; CRT, Circadian Reinforcement Therapy; CSQ-8, Client Satisfaction Questionnaire; ERSQ, Emotion Regulation Skills Questionnaire; ERSQ, Emotion Regulation Skills Questionnaire; EUROHIS-QOL-8, shortened version of the World Health Organization Quality of Life Instrument-Abbreviated Version (WHOQOL-BREF); FAST, Functioning Assessment Short Test; FMI, Freiburg Mindfulness Inventory; GAD-7, General Anxiety Disorder-7; GAF, Global Assessment of Functioning; GSES, General Self-Efficacy Scale; HEALTH-49, Hamburg Modules for the Assessment of Psychosocial Health; HDRS/HAM-D17, Hamilton Depression Rating Scale; IDS-SR, Inventory of Depressive Symptomatology Self Report; IG, intervention group; IPAQ, International Physical Activity Questionnaire; LIFE, Longitudinal Interval Follow-up Evaluation; MADRS-S, Montgomery-Asberg Depression Rating Scale; MDB, Monsenso Daybuilder; MDI, Major Depression Inventory; MEQ, Morningness-Eveningness Questionnaire; OCDS, Obsessive Compulsive Drinking Scale; PANAS, The Positive and Negative Affect Schedule; PHQ-9, Patient Health Quotient-9; PSR, Psychiatric Status Rating; PSQI, Pittsburgh Sleep Quality Index; PSS, Perceived stress scale; PTQ, Perseverative Thinking Questionnaire; QLDS, Quality of Life in Depression Scale; QOLI, Quality of Life Inventory; RDQ, Remission of Depression Questionnaire; RRS, Ruminative Response Scale; RS, Resilience Scale; SCS-D, Self-Compassion Scale - German; SCID-I, The Structured Clinical Interview for DSM; SCIP, Screen for Cognitive Impairment in Psychiatry; SCL-20, Symptom Checklist Depression Scale-20; SF-12, Short Form-12 Health Survey; SF-36, Short Form-36 Health Survey; STAI, State and Trait Anxiety Inventory; SUS, System Usability Scale; TAU, treatment as usual; TLFB, Timeline Follow Back; WHO-5, World Health Organization Well-Being Index; WL, waiting list; WHODAS 2.0, WHO Disability Assessment Schedule, Version 2.0; WHOQOL-BREF, World Health Organization Quality of Life Instrument-Abbreviated Version.

Table 2 presents a comprehensive summary of the results of the studies included in this analysis.

### 3.3 Quality assessment

We conducted a comprehensive risk of bias assessment for all 19 included studies using the Cochrane Risk of Bias Tool (Higgins et al., 2011). This tool evaluates seven domains: random sequence generation, allocation concealment, blinding of participants and personnel, blinding of outcome assessment, incomplete outcome data, selective reporting, and other biases (see Table 3). Random sequence generation was adequately reported in 78.9% (15/19) of the studies, employing methods such as computer-generated randomization, thus demonstrating a low risk of bias. However, 21.1% (4/19) of studies provided insufficient information, resulting in an unclear risk assessment. Allocation concealment procedures were less consistently reported. Only 31.6% (6/19) of studies clearly described appropriate methods, while 68.4% (13/19) lacked sufficient detail, leading to an unclear risk of bias assessment in this domain. Blinding presented a significant challenge, as is often the case with technology-based interventions. Double-blinding was reported in only one study, while 57.9% (11/19) implemented single-blind designs, typically involving blinded outcome assessors. The remaining 36.8% (7/19) did not specify blinding procedures, resulting in an unclear risk assessment. Outcome assessment blinding was adequately reported in 52.6% (10/19) of studies, indicating a low risk of bias. The other 47.4% (9/19) provided insufficient information, raising concerns about potential bias in this domain. Regarding incomplete outcome data, 84.2% (16/19) of studies demonstrated appropriate handling of attrition and missing data, indicating a low risk of bias. However, 15.8% (3/19) were assessed as having a high risk of bias in this area. All studies (19/19) showed a low risk of bias for selective reporting, suggesting consistent reporting of pre-specified outcomes across the research. Other potential sources of bias were unclear in 68.4% (13/19) of studies, primarily due to insufficient information about factors such as baseline imbalances or potential contamination between groups. The remaining 31.6% (6/19) were judged to have a low risk of other biases.

**Table 3:** Risk of bias assessment.

Study	Randomization method	Allocation concealment	Blinding	1	2	3	4
Vicent-Gil et al. (2022)	Block	NS	Single-blind (assessors blinded)	+	-	+	?
Aggestrup et al. (2023)	Computer-generated random list without stratification	NS	Single-blinded (rater)	+	+	+	?
Agyapong et al. (2012)	Computer-generated random list	NS	Single-blind (rater-blinded)	?	+	+	+
Kraft et al. (2017)	Centralized online procedure	Centralized randomization	NS	?	+	+	?
van den Berg et al. (2015)	NS	NS	NS	?	+	+	+
Browning et al. (2012)	Computer-generated random list	Blinded administrative staff selected shuffled consent forms	Single-blind (outcome assessors blinded)	?	+	+	?
Kok et al. (2015)	Computer-generated random list	Independent researcher conducted randomization	Single-blind (interviewers assessing outcomes were blinded)	+	+	+	?
Kordy et al. (2016)	Centralized online procedure	Centralized randomization	Evaluator-blind	+	+	+	+
Simon et al. (2011)	Automated random number generator, no blocking or stratification	Automated allocation	Treating physicians blinded to participation/allocation	?	+	+	+
Nyström et al. (2017)	Block (using computer software)	NS	NS	?	+	+	?
Zwerenz et al. (2017)	Block (using computer software)	NS	NS	+	-	+	?
Klein et al. (2018)	Simple randomization using Computer-generated random list	Independent researcher conducted randomization	Single-blind (interviewers assessing outcomes were blinded)	+	+	+	?
O’Reilly et al., 2019	Random number generator	NS	Assessor-blinded	+	+	+	?
Schlicker et al. (2017)	Allocation based on week of discharge	NS	NS	?	-	+	+
Segal et al. (2020)	Computer-generated random list using REDCap	Centralized randomization	Single-blind (assessors blinded)	?	+	+	+
Ebert et al. (2013)	Blindly drawing a random sample from shuffled consent forms	NS	NS	?	+	+	?
Hunkeler et al. (2012)	Random treatment assignments in blocked sets of four	Statistician blind to candidates’ identities prepared assignments	Telephone interviewers blind to treatment group	+	+	+	?
Holländare et al. (2013)	NS	NS	Not fully blinded (psychologist became aware of allocation during some interviews)	?	+	+	?
Hoorelbeke et al. (2015)	Automated randomization software	Sealed envelopes	Double-blind (participants and researchers)	+	+	+	?

1 = Blinding of outcome assessment; 2 = Incomplete outcome reporting; 3 = Selective outcome reporting; 4 = Other sources of bias; NS = Not specified; “+” = low risk; “-” = high risk; “?” = unclear risk.

Table 3 presents detailed ratings of the risk of bias domain for each of the studies included in this analysis.

### 3.4 Effects of the interventions

#### 3.4.1 Symptom severity

##### 3.4.1.1 Severity of anxiety symptoms

Six studies reported outcomes related to anxiety symptom severity. Aggestrup et al. (2023) found that the Circadian Reinforcement Therapy (CRT) group had significantly lower anxiety scores compared to the treatment as usual group, as measured by the HAM-D17. van den Berg et al. (2015) reported significantly lower anxiety scores in the telephone plus text message intervention group compared to the control group, with a difference of −2.04 points on the BSI-18 (*p* = 0.042). Browning et al. (2012) found that the Positive Face-based Attentional Bias Modification (ABM) intervention reduced anxiety symptoms during follow-up compared to placebo (*p* = 0.03), as measured by the STAI. Segal et al. (2020) observed a significantly greater reduction in GAD-7 scores in the Mindful Mood Balance plus usual depression care (MMB + UDC) group compared to usual care alone (mean difference 1.21, *p* = 0.004). The results of internet-based treatment interventions (Nyström et al., 2017), as measured by GAD-7, indicated a steeper decline in anxiety scores for the treatment group compared to the control group, with a between-group difference quantified as *B* = −0.384 (*p* = 0.023). Zwerenz et al. (2017) evaluated an internet-based intervention designed to reduce anxiety symptoms. Anxiety was measured using the GAD-7, and the findings revealed a significant reduction in anxiety symptoms for the intervention group compared to the wait-list control group, with scores of 11.06 ± 6.49 versus 13.15 ± 5.89, respectively (*p* = 0.02).

Overall, all six studies that reported anxiety outcomes found significant reductions in anxiety symptoms following digital interventions. However, the interventions and measurement tools varied considerably across studies, making direct comparisons challenging.

##### 3.4.1.2 Severity of depressive symptoms

Depressive symptom severity was the most reported outcome, with 9 out of 11 studies reporting significant improvements following digital interventions (Aggestrup et al., 2023; Agyapong et al., 2012; Holländare et al., 2011; Hunkeler et al., 2012; Kok et al., 2015; Nyström et al., 2017; Schlicker et al., 2017; Segal et al., 2020; Simon et al., 2011). Effect sizes ranged from small to large, indicating the potential for meaningful clinical impact. Short-term effects (3 months) were demonstrated by studies such as O’Reilly et al. (2019) and Kok et al. (2015), indicating that these interventions can provide rapid benefits. More importantly, long-term effects were observed in studies by Hunkeler et al. (2012) and Segal et al. (2020), with improvements maintained at 15 and 24 months, respectively.

Aggestrup et al. (2023) reported significantly lower HAM-D17 scores in the CRT (intervention) group compared to treatment as usual (estimated endpoint scores: CRT: 12.8, TAU: 13.6; *p* = 0.04). Agyapong et al. (2012) found significantly lower BDI-II scores in the supportive text message intervention group compared to controls at 3 months (8.5 vs. 16.7, *p* = 0.003). Kok et al. (2015) observed a significantly greater decrease in depressive symptoms measured by the IDS-SR30 in the mobile cognitive therapy group compared to treatment as usual (difference: −1.60 points per month, *p* = 0.003, Cohen’s *d* = 0.44 at 3 months). Simon et al. (2011) found significantly lower scores on depression measured using SCL-20, in the collaborative care management intervention group compared to usual care (0.95 vs. 1.17, *p* = 0.043, effect size 0.29). Nyström et al. (2017) reported significant reductions in PHQ-9 scores for the pooled treatment groups compared to control (*B* = -0.669, *p* = 0.001), with effect sizes (Hedges g) ranging from 1.30 to 2.36 for different intervention types. Schlicker et al. (2017) found a significantly smaller increase in BDI-II scores for the standardized text message intervention group compared to waitlist control (*p* < 0.05, Cohen’s *d* = 0.32). Segal et al. (2020) reported a significantly greater reduction in PHQ-9 scores in the MMB + UDC group compared to usual care alone (mean difference 0.95, *p* < 0.02). Hunkeler et al. (2012) found a greater reduction in depression severity for the eCare intervention group compared to usual care (estimate = −0.74 on a 6-point scale, *p* = 0.025, Cohen’s *d* = 0.60). However, Klein et al. (2018) and Zwerenz et al. (2017) found no significant differences in depressive symptom severity between the intervention and control groups. Holländare et al. (2013) reported a trend towards a larger decrease in depressive symptoms on the internet-based CBT group over time, with small to moderate effect sizes (Cohen’s *d* = 0.36 for BDI-II, *d* = 0.03 for MADRS-S at 24-month follow-up).

#### 3.4.2 Relapse and rehospitalization

Five studies examined the impact of digital interventions on relapse or recurrence rates, with mixed results. Holländare et al. (2013) found a substantial difference in relapse rates at 24 months, with significantly fewer relapses on the internet-based cognitive behavioral therapy (iCBT) group (13.7%) compared to the control group (60.9%). They also reported significantly higher remission rates in the iCBT group at 24 months. In contrast, Klein et al. (2018) found no significant difference in time to relapse between the intervention, M-CT group and TAU (hazard ratio = 0.77, 95% CI 0.53–1.14, *p* = 0.190). Similarly, there were no significant differences in the number of relapses between the intervention and control groups. Kordy et al. (2016) used a different metric, reporting on “unwell weeks.” They found that the internet-delivered SUMMIT intervention was associated with fewer “unwell weeks” compared to TAU (odds ratio = 0.48, 95% CI 0.23–0.98). However, the SUMMIT-PERSON intervention did not show a significant difference (odds ratio = 0.62, 95% CI 0.31–1.24).

#### 3.4.3 General quality of life and related functional measures

Four studies reported outcomes related to general quality of life or related functional measures, with mixed results. Zwerenz et al. (2017) employed the EUROHIS-QOL-8 to measure quality of life. At the 10-week follow-up, the intervention group demonstrated significantly higher EUROHIS-QOL-8 scores (mean ± SD: 2.15 ± 0.88) compared to the control group (1.87 ± 0.66). This difference was statistically significant (*p* = 0.04), with an effect size (Cohen’s d) of 0.53. Hunkeler et al. (2012) utilized the SF-36 Mental Health scale. They reported a significant improvement in the eCare group compared to the usual care group over the 24-month study period (*p* = 0.002). Vicent-Gil et al. (2022) used the Functioning Assessment Short Test (FAST) to assess psychosocial functioning, which is closely related to quality of life. At the 6-month follow-up, the authors reported significantly better functioning in the (intervention) INCREM group (mean ± SD: 9.33 ± 7.35) compared to the Psychoeducation group (24.11 ± 15.53). This difference was statistically significant (*p* = 0.041). Segal et al. (2020) found significantly greater improvement in mental functioning as measured by the SF-12 in the Mindful Mood Balance plus usual depression care (MMB + UDC) group compared to usual care alone (mean difference 5.10, *p* < 0.001). Hunkeler et al. (2012) reported greater improvement in SF-36 scores for the eCare intervention group compared to usual care (*p* = 0.002). Segal et al. (2020) used the SF-12 as a measure of mental functioning. At the 15-month follow-up, they found significantly better scores in the Mindful Mood Balance (MMB) plus Usual Depression Care (UDC) group compared to the UDC alone group. The between-group difference was −5.10 (SE 1.37), which was statistically significant (*p* < 0.001).

#### 3.4.4 Intervention adherence and engagement

Adherence to digital interventions, encompassing both primary treatment and aftercare phases, was reported in 11 of the 19 included studies. The methods of reporting and the rates of adherence varied considerably across studies.

##### 3.4.4.1 Primary intervention adherence

Completion rates for primary interventions showed substantial variability. Vicent-Gil et al. (2022) reported a 50% completion rate for their INCREM (intervention) program, which consisted of 12 weeks of cognitive and functional remediation. In contrast, Aggestrup et al. (2023) noted higher adherence, with 81.6% of participants completing the 4-week Circadian Reinforcement Therapy intervention. This difference might be attributed to the shorter duration of the latter intervention. Module or session completion rates also varied across studies: Kok et al. (2015) reported a mean completion of 5.5 out of 8 modules in the mobile cognitive therapy intervention over 8 weeks. Holländare et al. (2013) found higher completion rates, with participants finishing an average of 7.8 out of 9 mandatory modules in the 10-week internet-based CBT program. Zwerenz et al. (2017) reported that 86% of participants completed at least 6 out of 8 units in the 10-week psychodynamic web-based intervention.

Some studies provided more granular adherence data. Simon et al. (2011) reported that 78% of intervention participants completed at least one online care management contact, with a mean of 2.4 out of 3 planned contacts completing over 5 months. Segal et al. (2020) found that in the 8-session Mindful Mood Balance program, 70% of participants completed at least 4 sessions, while 54% completed all 8 sessions.

##### 3.4.4.2 Aftercare intervention adherence

For aftercare interventions, adherence patterns often showed a decline over time. Kordy et al. (2016) reported on the 12-month SUMMIT program, noting that 70% of participants in the SUMMIT group and 76% in the SUMMIT-PERSON group remained active for at least 80% of the intervention period. However, engagement decreased over time, with the median number of website visits declining from 13 in the first month to 2 in the final month. Hunkeler et al. (2012) observed a similar trend in the 12-month eCare aftercare program. Website usage dropped from 50% of participants in the first month to 30% by month 24. In contrast, Klein et al. (2018) reported more stable adherence in the 24-month mobile cognitive therapy aftercare intervention. The results showed that 85% of participants completed at least 5 out of 8 modules, with an average of 6.6 modules completed.

##### 3.4.4.3 Engagement with specific intervention components

Some studies reported on engagement with aspects of the interventions. O’Reilly et al. (2019) noted that participants read an average of 83.3% of the supportive text messages sent over a 6-month period, although they did not distinguish between primary and aftercare phases. Hunkeler et al. (2012), in addition to website usage, reported on other components of the eCare program. The results highlighted that 39% of participants used the online discussion group, and 36% used the secure messaging feature to communicate with care managers.

It’s important to note that 8 out of 19 studies (Agyapong et al., 2012; Browning et al., 2012; Ebert et al., 2013; Hoorelbeke et al., 2015; Klein et al., 2018; Kraft et al., 2017; Nyström et al., 2017; van den Berg et al., 2015) did not report specific adherence data.

The variability in adherence reporting and metrics used across studies reflects the heterogeneity of digital interventions for depression. While adherence rates were generally acceptable for both primary and aftercare interventions, there was substantial variability across studies. A common trend emerged of decreasing engagement over time, particularly in longer interventions, although some studies managed to maintain more stable adherence rates.

Relapse rates and symptom severity were the primary outcomes analyzed. Additional relevant findings, including effects on physiological, cognitive, and psychosocial parameters, are reported in the Supplementary material S2.

## 4 Discussion

### 4.1 Synthesis and theoretical implications

This systematic review synthesized evidence from 19 randomized controlled trials examining the efficacy of digital interventions for depression and anxiety aftercare, revealing several key theoretical pathways through which these interventions may influence mental health outcomes. Of the included studies, 14 reported significant improvements in depressive symptoms (effect sizes ranging from small to large, Cohen’s *d* = 0.20–0.80), while only 6 studies assessed anxiety outcomes, highlighting a theoretical gap in understanding transdiagnostic effects. The variation in adherence rates (reported in 11 studies) and engagement patterns supports theoretical frameworks emphasizing the importance of human support in digital interventions (Torous et al., 2020a,b). Notably, guided interventions consistently showed higher completion rates compared to fully automated ones, with dropout rates varying from 7.7 to 50% across studies. These patterns, combined with the finding that only 4 studies assessed quality of life outcomes, suggest the need for more sophisticated theoretical models of digital intervention implementation that address both clinical efficacy and user engagement (Torous et al., 2020a,b).

The efficacy of digital interventions for depression appeared to operate through multiple theoretically-grounded mechanisms. Internet-based cognitive therapy (Kok et al., 2015) and mobile cognitive therapy (Klein et al., 2018) both demonstrated promise in reducing depressive symptoms through cognitive modification pathways, supporting cognitive theories of depression that emphasize the role of information processing in symptom maintenance (Bockting et al., 2015). Our findings align with the established efficacy of cognitive therapy in traditional face-to-face settings and corroborates findings from previous meta-analyses on internet-based cognitive behavioral therapy (iCBT) for depression (Andersson et al., 2014; Karyotaki et al., 2017; Karyotaki et al., 2021).

Mindfulness-based interventions, particularly the Mindful Mood Balance program (Segal et al., 2020), showed efficacy consistent with metacognitive models of depression (Spijkerman et al., 2016). These findings extend our understanding of how mindfulness mechanisms (specifically decentering and metacognitive awareness) can be effectively translated to digital formats, building on established theoretical frameworks of mindfulness-based cognitive therapy (Kuyken et al., 2016).

In the context of anxiety symptoms, only 6 out of the 19 studies provided clear data on this domain. Digital interventions were shown to operate through complementary theoretical mechanisms. Notably, the effectiveness of the Mindful Mood Balance program in reducing anxiety symptoms (Segal et al., 2020) supports transdiagnostic theories of emotional regulation in anxiety and depression (Sauer-Zavala et al., 2020). Additionally, multi-component interventions combining telephone contact and text messages (van den Berg et al., 2015) align with theoretical models emphasizing the importance of multiple channels of support in anxiety management (Craske and Stein, 2016). However, given the limited number of anxiety assessments across studies, further conclusions on the theoretical pathways for the efficacy of digital interventions remain challenging. Regarding relapse prevention, this systematic review identified five studies that specifically addressed relapse or recurrence rates. The interventions that showed promise in reducing relapse rates, such as internet-based cognitive behavioral therapy (iCBT) (Holländare et al., 2013) and mobile cognitive therapy (M-CT) (Klein et al., 2018), share a foundation in cognitive-behavioral principles. These findings support the role of ongoing skill practice and monitoring in maintaining therapeutic gains (Bockting et al., 2018). However, the variability in effect sizes—from highly significant to statistically non-significant – raises questions about other factors that may moderate intervention efficacy.

For example, the effectiveness of the SUMMIT program, which led to an increase in “well weeks” over a 24-month period (Kordy et al., 2016) aligns with theoretical framework conceptualizing depression as a chronic, recurrent condition, which requires continuous support in maintaining recovery (Bockting et al., 2015). However, it also raises questions about the optimal duration and intensity of digital interventions, and how they might be integrated into stepped care approaches. The tendency for more positive results in studies with longer follow-up periods (e.g., 24 months) suggests that the benefits of these interventions may accumulate over time, possibly by helping patients internalize coping strategies or by providing a safety net during vulnerable periods.

Our systematic review revealed that only 4 out of 19 studies specifically assessed quality of life outcomes. Quality of life improvements across psychodynamic, disease management, and mindfulness-based interventions (Zwerenz et al., 2017; Hunkeler et al., 2012; Segal et al., 2020) align with the broader literature suggesting that internet-based interventions can improve quality of life in individuals with depression (Karyotaki et al., 2017; Karyotaki et al., 2021). These results support theoretical frameworks looking at the interconnection between symptom improvement and functional recovery, and more than that, they emphasize the importance of functional recovery as a primary goal in treating major depressive disorder, advocating for clinical assessments that go beyond symptom alleviation to address work, social, and overall life functionality (Lam et al., 2015) Adherence rates (ranging from 50 to 92.3%) are consistent with previous evidence, which found dropout rates in smartphone app trials for depression ranging from 0 to 62.7%, with a pooled dropout rate of 31.3% (Torous et al., 2020a,b). These results highlight the importance of human support in digital interventions (Mohr et al., 2019), with guided interventions showing higher completion rates.

Our synthesis suggests that digital interventions operate through multiple theoretical pathways, including cognitive modification, behavioral activation, emotional regulation, and social support mechanisms. Effectiveness appears to be moderated by factors such as intervention design, level of human support, and individual patient characteristics, suggesting the need for more sophisticated theoretical models of digital intervention implementation and personalization (Torous et al., 2021).

### 4.2 Implementation strategies and clinical practice guidelines

The integration of digital interventions into existing care pathways reveals both design opportunities and implementation complexities. Anaysis of intervention design features showed that shorter programs generally achieved higher adherence rates. For example, Aggestrup et al.’s (2023) 4-week intervention achieved 81.6% completion, contrasting lower adherence rates in longer programs (e.g.: Vicent-Gil et al., 2022). This inverse relationship between duration and adherence aligns with findings from the broader digital health literature (Kelders et al., 2012). The level of human support emerged as crucial - guided interventions, such as the therapist-supported program in Holländare et al. (2013), demonstrated higher completion rates compared to full automated ones. This observation is consistent with meta-analytic findings suggesting that guided internet-based interventions yield larger effect sizes and better adherence than unguided ones (Baumeister et al., 2014). Furthermore, the mode of delivery significantly impacted engagement patterns, with text message-based interventions (Agyapong et al., 2012; O’Reilly et al., 2019), showing high engagement rates (possibly due to low burden and daily life integration), while complex web-based platforms showed variable adherence.

Integration strategies varied significantly across studies. While some interventions, like Kordy et al.'s (2016) SUMMIT program, functioned as stand-alone aftercare interventions, others, such as Agyapong et al.'s (2012) text message intervention, served as adjuncts to standard care. This diversity highlights the need for flexible integration approaches. Studies like Simon et al. (2011) and Hunkeler et al. (2012) demonstrated how digital tools can effectively complement usual care, though successful implementation required substantial provider support and training. The role of healthcare providers proved critical, i.e., studies where guided interventions were consistently incorporated showed better outcomes and high adherence rates, ranging from 76.2 to 96.1% (Hunkeler et al., 2012; Holländare et al., 2013).

Several implementation barriers emerged across studies. Data integration and interoperability presented significant challenges - none of the reviewed studies successfully addressed the integration of intervention data into existing electronic health records. The potential to exacerbate health inequalities through digital divides was evident but inadequately addressed, particularly regarding accessibility for populations with limited technological literacy or access. Regulatory and ethical considerations, especially regarding data privacy and security, were not prominently featured in the studies despite their importance for implementation. Cost-effectiveness data was largely absent, with only limited evidence from Kordy et al. (2016) suggesting potential economic benefits.

Personalization emerged as a promising strategy for enhancing engagement. This was demonstrated in the SUMMIT-PERSON intervention (Kordy et al., 2016), which showed improved adherence compared to equivalent non-personalized version. Engagement-promoting features such as reminders and progress tracking (Simon et al., 2011) showed potential benefits, though their relationship with clinical outcomes requires further investigation. The evidence suggests that successful implementation depends on matching intervention complexity and support levels to both healthcare context and patient characteristics, while ensuring adequate provider training and clear integration protocols (Hornstein et al., 2021).

For clinical practice, our findings provide several actionable recommendations. First, the evidence supports using digital interventions specifically for preventing depressive relapse, with studies like Holländare et al. (2013) and Klein et al. (2018) demonstrating significant reductions in relapse rates over extended follow-up periods. Second, intervention selection should be tailored to patient characteristics – e.g., the Circadian Reinforcement Therapy showed particular benefit for patients with circadian rhythm disturbances (Aggestrup et al., 2023), while cognitive control training benefited those with persistent rumination (Hoorelbeke et al., 2015). Third, to address adherence challenges, clinicians should prioritize interventions incorporating regular support or guidance, exemplified by the therapist-supported internet-based cognitive therapy (Kok et al., 2015). The high satisfaction rates reported in studies like Zwerenz et al. (2017) and Simon et al. (2011) indicate that patients find digital interventions acceptable when properly supported and integrated into their care plan. For successful implementation, clinicians should: assess patient suitability based on technological literacy and access; select interventions matching existing care pathways; ensure adequate monitoring systems; maintain flexibility in delivery based on patient response. This stepped approach to implementation, supported by regular outcome monitoring and adjustment, may optimize the potential benefits while minimizing dropout risks.

### 4.3 Methodological considerations and future directions

The review of digital interventions for depression management and relapse prevention revealed several methodological considerations and limitations that inform future research directions.

The heterogeneity of studies posed a significant challenge, with intervention types ranging from text message-based systems (Agyapong et al., 2012) to complex web-based platforms (Kordy et al., 2016). This variability in intervention design, combined with differences in duration, intensity of human support, and theoretical frameworks, further limited our ability to draw definitive conclusions about optimal implementation strategies.

Control conditions presented another methodological concern, with most studies employing treatment as usual or waitlist controls, rather than active comparators. The predominance of non-active control conditions limits our understanding of specific intervention effects versus non-specific engagement benefits. The lack of attention-placebo controls in digital intervention research particularly affects our ability to distinguish between therapeutic components and general benefits of technological engagement.

Outcome measurement heterogeneity further complicated cross-study comparisons. While depression measures were consistently reported, the diversity of instruments used (e.g., BDI-II, HDRS, PHQ-9) and inconsistent reporting of secondary outcomes, particularly anxiety symptoms and quality of life measures, hindered comprehensive effectiveness evaluation. Furthermore, the duration of follow-up varied widely, from immediate post-intervention assessments to 24-month follow-ups, which limits our interpretation of long-term effects.

Sample sizes and statistical power were concerns in several studies. While some larger trials provided robust evidence (e.g., Segal et al., 2020), many studies had relatively small sample sizes, limiting detection of meaningful effects in secondary outcomes or subgroup analyses.

In addition, the inconsistent reporting of adherence and engagement metrics further complicated interpretation, with many studies failing to provide detailed information on participants’ interaction with interventions.

With respect to generalizability, issues pertaining to selection bias were evident across studies, with recruitment conducted primarily through online platforms or specialized clinics, potentially excluding broader population segments. Individuals with severe depression, suicidal ideation, or comorbid conditions were frequently excluded, which further limits generalizability. Additionally, the rapid pace of technological advancement means that the study interventions might be outdated by publication time, a unique challenge in digital health research.

These methodological considerations point to three priority directions for future research. First, mechanism-focused studies are needed to elucidate how digital interventions affect change, particularly examining specific therapeutic components versus non-specific effects. This review identified several promising mechanisms, which may warrant further investigation, including cognitive processes (Browning et al., 2012; Zwerenz et al., 2017), emotion regulation (Hoorelbeke et al., 2015) and behavioural activation patterns. Second, implementation research should focus on optimizing intervention delivery through personalization algorithms and adaptation to individual patient characteristics. Finally, larger-scale trials with active control conditions and longer follow-up periods are needed to establish comparative efficacy and sustainability of treatment effects.

## 5 Conclusion

This systematic review offers novel insights into internet- and mobile-based aftercare and relapse prevention interventions for anxiety and depressive disorders. It synthesizes evidence on both internet- and mobile-based interventions specifically for post-acute care, addressing a critical literature gap. The transdiagnostic approach discussed here and the inclusion of long-term follow-up studies provide perspectives on intervention efficacy across comorbid conditions and over extended periods.

A key contribution is the identification of adherence and long-term engagement as critical success factors, highlighting the need for effective engagement strategies. Our examination of integration challenges in existing care pathways provides guidance for implementation. By critiquing current methodological limitations and proposing standardized approaches, we suggest a new standard for future research. Furthermore, our synthesis of novel research directions, including personalized interventions and emerging technologies, provides a roadmap for advancing the field.

This systematic review consolidates current knowledge and contributes to the field by identifying critical gaps, methodological improvements, and future research priorities. These insights have the potential to guide the development of more effective, accessible, and personalized digital aftercare interventions, ultimately improving long-term outcomes for individuals with anxiety and depressive disorders.

## Data Availability

The original contributions presented in the study are included in the article/Supplementary material, further inquiries can be directed to the corresponding author.

## References

[ref1] Aggestrup A. S. Svendsen S. D. Præstegaard A. Løventoft P. Nørregaard L. Knorr U. . (2023). Circadian reinforcement therapy in combination with electronic self-monitoring to facilitate a safe Postdischarge period for patients with major depression: randomized controlled trial. JMIR Ment Health 10:e50072. doi: 10.2196/50072, PMID: 37800194 PMC10714270

[ref2] Agyapong V. I. O. Ahern S. McLoughlin D. M. Farren C. K. (2012). Supportive text messaging for depression and comorbid alcohol use disorder: single-blind randomised trial. J. Affect. Disord. 141, 168–176. doi: 10.1016/j.jad.2012.02.040, PMID: 22464008

[ref3] Andersson G. Cuijpers P. Carlbring P. Riper H. Hedman E. (2014). Guided internet-based vs. face-to-face cognitive behavior therapy for psychiatric and somatic disorders: a systematic review and meta-analysis. World Psychiatry 13, 288–295. doi: 10.1002/wps.20151, PMID: 25273302 PMC4219070

[ref6] Arias D. Saxena S. Verguet S. (2022). Quantifying the global burden of mental disorders and their economic value. EClinicalMedicine 54:101675. doi: 10.1016/j.eclinm.2022.101675 36193171 PMC9526145

[ref9001] Baumeister H. Nowoczin L. Lin J. Seifferth H. Seufert J. Laubner K. . (2014). Impact of an acceptance facilitating intervention on diabetes patients’ acceptance of Internet-based interventions for depression: a randomized controlled trial. Diabetes Res. Clin. Pract. 105, 30–39. doi: 10.1016/j.diabres.2014.04.031 24862240

[ref8] Bockting C. L. H. Klein N. S. Elgersma H. J. van Rijsbergen G. D. Slofstra C. Ormel J. . (2018). Effectiveness of preventive cognitive therapy while tapering antidepressants versus maintenance antidepressant treatment versus their combination in prevention of depressive relapse or recurrence (DRD study): a three-group, multicentre, randomised controlled trial. Lancet Psychiatry 5, 401–410. doi: 10.1016/S2215-0366(18)30100-7, PMID: 29625762

[ref7] Bockting C. L. Hollon S. D. Jarrett R. B. Kuyken W. Dobson K. (2015). A lifetime approach to major depressive disorder: the contributions of psychological interventions in preventing relapse and recurrence. Clin. Psychol. Rev. 41, 16–26. doi: 10.1016/j.cpr.2015.02.003, PMID: 25754289

[ref9] Browning M. Holmes E. A. Charles M. Cowen P. J. Harmer C. J. (2012). Using attentional bias modification as a cognitive vaccine against depression. Biol. Psychiatry 72, 572–579. doi: 10.1016/j.biopsych.2012.04.014, PMID: 22579509 PMC3504298

[ref10] Craske M. G. Stein M. B. (2016). Anxiety. Lancet 388, 3048–3059. doi: 10.1016/S0140-6736(16)30381-6 27349358

[ref12] Ebert D. D. Van Daele T. Nordgreen T. Karekla M. Compare A. Zarbo C. . (2018). Internet- and Mobile-based psychological interventions: applications, efficacy, and potential for improving mental health A report of the EFPA E-health taskforce. Eur. Psychol. 23, 167–187. doi: 10.1027/1016-9040/a000318

[ref11] Ebert D. Tarnowski T. Gollwitzer M. Sieland B. Berking M. (2013). A transdiagnostic internet-based maintenance treatment enhances the stability of outcome after inpatient cognitive behavioral therapy: a randomized controlled trial. Psychother. Psychosom. 82, 246–256. doi: 10.1159/000345967, PMID: 23736751

[ref15] Gold S. M. Köhler-Forsberg O. Moss-Morris R. Mehnert A. Miranda J. J. Bullinger M. . (2020). Comorbid depression in medical diseases. Nat. Rev. Dis. Prim. 6:69. doi: 10.1038/s41572-020-0200-2 32820163

[ref17] Hennemann S. Farnsteiner S. Sander L. (2018). Internet- and mobile-based aftercare and relapse prevention in mental disorders: A systematic review and recommendations for future research. Internet Interv. 14, 1–17. doi: 10.1016/j.invent.2018.09.001, PMID: 30510909 PMC6205252

[ref18] Higgins J. P. T. Altman D. G. Gøtzsche P. C. Jüni P. Moher D. Oxman A. D. . (2011). The Cochrane Collaboration’s tool for assessing risk of bias in randomised trials. BMJ 343:d5928. doi: 10.1136/bmj.d5928 22008217 PMC3196245

[ref20] Holländare F. Anthony S. Randestad M. Tillfors M. Carlbring P. Andersson G. . (2013). Two-year outcome of internet-based relapse prevention for partially remitted depression. Behav. Res. Ther. 51, 719–722. doi: 10.1016/j.brat.2013.08.002, PMID: 24021360

[ref21] Holländare F. Johnsson S. Randestad M. Tillfors M. Carlbring P. Andersson G. . (2011). Randomized trial of internet-based relapse prevention for partially remitted depression. Acta Psychiatr. Scand. 124, 285–294. doi: 10.1111/j.1600-0447.2011.01698.x, PMID: 21401534

[ref22] Hoorelbeke K. Faelens L. Behiels J. Koster E. H. W. (2015). Internet-delivered cognitive control training as a preventive intervention for remitted depressed patients: protocol for a randomized controlled trial. BMC Psychiatry 15:125. doi: 10.1186/s12888-015-0511-0, PMID: 26055122 PMC4459439

[ref23] Hornstein S. Forman-Hoffman V. Nazander A. Ranta K. Hilbert K. (2021). Predicting therapy outcome in a digital mental health intervention for depression and anxiety: A machine learning approach. Digit. Health 7:659. doi: 10.1177/20552076211060659 PMC8637697 34868624

[ref24] Huijbers M. J. Spinhoven P. Spijker J. Ruhé H. G. Van Schaik D. J. F. Van Oppen P. . (2015). Adding mindfulness-based cognitive therapy to maintenance antidepressant medication for prevention of relapse/recurrence in major depressive disorder: randomised controlled trial. J. Affect. Disord. 187, 54–61. doi: 10.1016/j.jad.2015.08.023 26318271

[ref25] Hunkeler E. M. Hargreaves W. A. Fireman B. Terdiman J. Meresman J. F. Porterfield Y. . (2012). A web-delivered care management and patient self-management program for recurrent depression: a randomized trial. Psychiatr. Serv. 63, 1063–1071. doi: 10.1176/appi.ps.005332011, PMID: 22983558

[ref26] Karyotaki E. Efthimiou O. Miguel C. Bermpohl F. M. G. Furukawa T. A. Cuijpers P. . (2021). Internet-based cognitive behavioral therapy for depression: A systematic review and individual patient data network Meta-analysis. JAMA Psychiatry 78, 361–371. doi: 10.1001/jamapsychiatry.2020.4364, PMID: 33471111 PMC8027916

[ref27] Karyotaki E. Riper H. Twisk J. Hoogendoorn A. Kleiboer A. Mira A. . (2017). Efficacy of self-guided internet-based cognitive behavioral therapy in the treatment of depressive symptoms: A Meta-analysis of individual participant data. JAMA Psychiatry 74, 351–359. doi: 10.1001/jamapsychiatry.2017.0044, PMID: 28241179

[ref28] Kelders S. M. Kok R. N. Ossebaard H. C. Van Gemert-Pijnen J. E. W. C. (2012). Persuasive system design does matter: a systematic review of adherence to web-based interventions. J. Med. Internet Res. 14:e152. doi: 10.2196/jmir.2104, PMID: 23151820 PMC3510730

[ref29] Klein N. S. Kok G. D. Burger H. van Valen E. Riper H. Cuijpers P. . (2018). No sustainable effects of an internet-based relapse prevention program over 24 months in recurrent depression: primary outcomes of a randomized controlled trial. Psychother. Psychosom. 87, 55–57. doi: 10.1159/000485039 29306953

[ref30] Kok G. Bockting C. Burger H. Smit F. Riper H. (2014). Mobile cognitive therapy: adherence and acceptability of an online intervention in remitted recurrently depressed patients. Internet Interv. 1, 65–73. doi: 10.1016/j.invent.2014.05.002

[ref31] Kok G. Burger H. Riper H. Cuijpers P. Dekker J. van Marwijk H. . (2015). The three-month effect of mobile internet-based cognitive therapy on the course of depressive symptoms in remitted recurrently depressed patients: results of a randomized controlled trial. Psychother. Psychosom. 84, 90–99. doi: 10.1159/000369469, PMID: 25721915

[ref32] Kordy H. Wolf M. Aulich K. Bürgy M. Hegerl U. Hüsing J. . (2016). Internet-delivered disease management for recurrent depression: A multicenter randomized controlled trial. Psychother. Psychosom. 85, 91–98. doi: 10.1159/000441951 26808817

[ref33] Kraft S. Wolf M. Klein T. Becker T. Bauer S. Puschner B. (2017). Text message feedback to support mindfulness practice in people with depressive symptoms: A pilot randomized controlled trial. JMIR Mhealth Uhealth 5:e59. doi: 10.2196/mhealth.7095, PMID: 28465278 PMC5434251

[ref34] Kuyken W. Warren F. C. Taylor R. S. Whalley B. Crane C. Bondolfi G. . (2016). Efficacy of mindfulness-based cognitive therapy in prevention of depressive relapse: an individual patient data Meta-analysis from randomized trials. JAMA Psychiatry 73, 565–574. doi: 10.1001/jamapsychiatry.2016.0076, PMID: 27119968 PMC6640038

[ref35] Lam R. Parikh S. Michalak E. Dewa C. Kennedy S. (2015). Canadian network for mood and anxiety treatments (CANMAT) consensus recommendations for functional outcomes in major depressive disorder. Ann. Clin. Psychiatry 27, 142–149, PMID: 25954941

[ref37] Malhi G. S. Mann J. J. (2018). Depression. Lancet 392, 2299–2312. doi: 10.1016/S0140-6736(18)31948-2 30396512

[ref9002] Mohr D. C. Lattie E. G. Tomasino K. N. Kwasny M. J. Kaiser S. M. Gray E. L. . (2019). A randomized noninferiority trial evaluating remotely-delivered stepped care for depression using internet cognitive behavioral therapy (CBT) and telephone CBT. Behav. Res. Ther. 123:103485. doi: 10.1016/j.brat.2019.103485 31634738 PMC6916718

[ref40] Naslund J. A. Aschbrenner K. A. Araya R. Marsch L. A. Unützer J. Patel V. . (2017). Digital technology for treating and preventing mental disorders in low-income and middle-income countries: a narrative review of the literature. Lancet Psychiatry 4, 486–500. doi: 10.1016/S2215-0366(17)30096-2, PMID: 28433615 PMC5523650

[ref42] Nyström M. B. T. Stenling A. Sjöström E. Neely G. Lindner P. Hassmén P. . (2017). Behavioral activation versus physical activity via the internet: A randomized controlled trial. J. Affect. Disord. 215, 85–93. doi: 10.1016/j.jad.2017.03.018, PMID: 28319696

[ref43] O’Reilly H. Hagerty A. O’Donnell S. Farrell A. Hartnett D. Murphy E. . (2019). Alcohol use disorder and comorbid depression: A randomized controlled trial investigating the effectiveness of supportive text messages in aiding recovery. Alcohol Alcohol. 54, 551–558. doi: 10.1093/alcalc/agz060, PMID: 31361815

[ref45] Page M. J. Moher D. Bossuyt P. M. Boutron I. Hoffmann T. C. Mulrow C. D. . (2021). PRISMA 2020 explanation and elaboration: updated guidance and exemplars for reporting systematic reviews. BMJ 372:160. doi: 10.1136/bmj.n160 PMC8005925 33781993

[ref48] Sander L. Rausch L. Baumeister H. (2016). Effectiveness of internet- and mobile-based psychological interventions for the prevention of mental disorders: A systematic review and meta-analysis protocol. Syst. Rev. 5, 1–5. doi: 10.1186/s13643-016-0209-5 26880167 PMC4754873

[ref50] Sauer-Zavala S. Bentley K. H. Steele S. J. Tirpak J. W. Ametaj A. A. Nauphal M. . (2020). Treating depressive disorders with the unified protocol: A preliminary randomized evaluation. J. Affect. Disord. 264, 438–445. doi: 10.1016/j.jad.2019.11.072 31759672 PMC7024024

[ref51] Schlicker S. Ebert D. D. Middendorf T. Titzler I. Berking M. (2017). Evaluation of a text-message-based maintenance intervention for major depressive disorder after inpatient cognitive behavioral therapy. J. Affect. Disord. 227, 305–312. doi: 10.1016/j.jad.2017.10.047 29132073

[ref52] Schlief M. Saunders K. R. K. Appleton R. Barnett P. Vera San Juan N. Foye U. . (2022). Synthesis of the evidence on what works for whom in Telemental health: rapid realist review. Interact J Med Res 11:e38239. doi: 10.2196/38239, PMID: 35767691 PMC9524537

[ref54] Segal Z. V. Dimidjian S. Beck A. Boggs J. M. Vanderkruik R. Metcalf C. A. . (2020). Outcomes of online mindfulness-based cognitive therapy for patients with residual depressive symptoms: A randomized clinical trial. JAMA Psychiatry 77, 563–573. doi: 10.1001/jamapsychiatry.2019.4693, PMID: 31995132 PMC6990961

[ref55] Simon G. E. Ralston J. D. Savarino J. Pabiniak C. Wentzel C. Operskalski B. H. (2011). Randomized trial of depression follow-up care by online messaging. J. Gen. Intern. Med. 26, 698–704. doi: 10.1007/s11606-011-1679-8, PMID: 21384219 PMC3138593

[ref56] Solis E. C. van Hemert A. M. Carlier I. V. E. Wardenaar K. J. Schoevers R. A. Beekman A. T. F. . (2021). The 9-year clinical course of depressive and anxiety disorders: new NESDA findings. J. Affect. Disord. 295, 1269–1279. doi: 10.1016/j.jad.2021.08.108, PMID: 34706441

[ref57] Spijkerman M. P. J. Pots W. T. M. Bohlmeijer E. T. (2016). Effectiveness of online mindfulness-based interventions in improving mental health: A review and meta-analysis of randomised controlled trials. Clin. Psychol. Rev. 45, 102–114. doi: 10.1016/j.cpr.2016.03.009, PMID: 27111302

[ref9003] Torous J. Bucci S. Bell I. H. Kessing L. V. Faurholt-Jepsen M. Whelan P. . (2021). The growing field of digital psychiatry: current evidence and the future of apps, social media, chatbots, and virtual reality. World Psychiatry: Official Journal of the World Psychiatric Association (WPA), 20, 318–335. doi: 10.1002/wps.20883 34505369 PMC8429349

[ref58] Torous J. Lipschitz J. Ng M. Firth J. (2020a). Dropout rates in clinical trials of smartphone apps for depressive symptoms: A systematic review and meta-analysis. J. Affect. Disord. 263, 413–419. doi: 10.1016/j.jad.2019.11.167 31969272

[ref59] Torous J. Myrick K. J. Rauseo-Ricupero N. Firth J. (2020b). Digital mental health and COVID-19: using technology today to accelerate the curve on access and quality tomorrow. JMIR Ment Health 7:e18848. doi: 10.2196/18848, PMID: 32213476 PMC7101061

[ref60] van den Berg N. Grabe H.-J. Baumeister S. E. Freyberger H. J. Hoffmann W. (2015). A telephone- and text message-based telemedicine concept for patients with mental health disorders: results of a randomized controlled trial. Psychother. Psychosom. 84, 82–89. doi: 10.1159/000369468, PMID: 25721861

[ref9004] Vicent-Gil M. González-Simarro S. Raventós B. Vera J. Marín Martínez E. D. Sabaté-Cao C. . (2022). Randomized clinical trial of integral cognitive remediation program for major depression (INCREM). J. Affect. Disord. 310, 189–197. doi: 10.1016/j.jad.2022.05.016 35545155

[ref61] Vicent-Gil M. Raventós B. Marín-Martínez E. D. González-Simarro S. Martínez-Arán A. Bonnin C. D. M. . (2019). Testing the efficacy of INtegral cognitive REMediation (INCREM) in major depressive disorder: study protocol for a randomized clinical trial. BMC Psychiatry 19:135. doi: 10.1186/s12888-019-2117-4, PMID: 31060604 PMC6501398

[ref62] Zwerenz R. Becker J. Johansson R. Frederick R. J. Andersson G. Beutel M. E. (2017). Transdiagnostic, psychodynamic web-based self-help intervention following inpatient psychotherapy: results of a feasibility study and randomized controlled trial. JMIR Ment. Health 4:e41. doi: 10.2196/mental.7889 PMC5662790 29038094

