# Supplementary material for: Internet- and mobile-based aftercare and relapse prevention interventions for anxiety and depressive disorders: a systematic review
Source: Front Psychol. 2024 Dec 12;15:1474016. doi: 10.3389/fpsyg.2024.1474016 (PMC11670138; doi:10.3389/fpsyg.2024.1474016)
Supplement: Supplementary file 1 [file Data_Sheet_1.pdf]

Search strings

1. MEDLINE via PubMed

((((((((((((((((((((tertiary prevention[MeSH Terms]) OR secondary prevention[MeSH Terms]) OR aftercare[MeSH Terms]) OR aftercare[Title/Abstract]) OR after-care[Title/Abstract]) OR after-treatment*[Title/Abstract]) OR "after treatment*"[Title/Abstract]) OR "relapse prevention"[Title/Abstract]) OR "follow-up intervention"[Title/Abstract]) OR post-treatment*[Title/Abstract]) OR "post treatment*"[Title/Abstract]) OR "maintenance treatment*"[Title/Abstract]) OR "continuation treatment*"[Title/Abstract]) OR "post discharge"[Title/Abstract]) OR "tertiary prevention"[Title/Abstract]) OR "secondary prevention"[Title/Abstract]) OR post-discharge[Title/Abstract]) OR "continuity of care"[Title/Abstract])) AND ((((((((((((((((((((((((telemedicine[MeSH Terms]) OR Therapy, Computer-Assisted[MeSH Terms]) OR internet[MeSH Terms]) OR Mobile Applications[MeSH Terms]) OR online*[Title/Abstract]) OR computer*[Title/Abstract]) OR internet*[Title/Abstract]) OR web[Title/Abstract]) OR mobile*[Title/Abstract]) OR smartphone*[Title/Abstract]) OR web-based[Title/Abstract]) OR ehealth[Title/Abstract]) OR e-health[Title/Abstract]) OR mhealth[Title/Abstract]) OR m-health[Title/Abstract]) OR e-therapy[Title/Abstract]) OR telemental[Title/Abstract]) OR ICBT[Title/Abstract]) OR e-mail[Title/Abstract]) OR email[Title/Abstract]) OR SMS[Title/Abstract]) OR "text message*"[Title/Abstract]) OR text-message*[Title/Abstract]) OR messaging[Title/Abstract]) OR CCBT[Title/Abstract]) OR chat*[Title/Abstract])) AND ((((((((((((((((((depression[MeSH Terms]) OR anxiety[MeSH Terms]) OR anxiety disorders[MeSH Terms]) OR depressive disorder[MeSH Terms]) OR fear[MeSH Terms]) OR depressi*[Title/Abstract]) OR dysthymi*[Title/Abstract]) OR "Premenstrual dysphoric disorder"[Title/Abstract]) OR "mood disorder*"[Title/Abstract]) OR "affective disorder*"[Title/Abstract]) OR MDD[Title/Abstract]) OR anxiety[Title/Abstract]) OR anxious[Title/Abstract]) OR panic*[Title/Abstract]) OR phobi*[Title/Abstract]) OR GAD[Title/Abstract]) OR fear[Title/Abstract]) OR agoraphobi*[Title/Abstract])) AND (((((((randomized controlled trial[Publication Type]) OR controlled clinical trial[Publication Type]) OR randomized[Title/Abstract]) OR randomised[Title/Abstract]) OR randomly[Title/Abstract]) OR trial[Title/Abstract]) OR RCT[Text Word])

3. Scopus via e-nformation.ro

((TITLE-ABS-KEY (aftercare)) OR (TITLE-ABS-KEY ({after-care})) OR (TITLE-ABS-KEY ({after-treatment})) OR (TITLE-ABS-KEY ("after treatment*")) OR (TITLE-ABS-KEY ("relapse prevention")) OR (TITLE-ABS-KEY ({follow-up intervention})) OR (TITLE-ABS-KEY ({post-treatment})) OR (TITLE-ABS-KEY ("post treatment*")) OR (TITLE-ABS-KEY ("maintenance treatment*")) OR (TITLE-ABS-KEY ("continuation treatment*")) OR (TITLE-ABS-KEY ("post discharge")) OR (TITLE-ABS-KEY ("tertiary prevention")) OR (TITLE-ABS-KEY ("secondary prevention")) OR (TITLE-ABS-KEY ({post-discharge})) OR (TITLE-ABS-KEY ("continuity of care"))) AND ((TITLE-ABS-KEY (online*)) OR (TITLE-ABS-KEY (computer*)) OR (TITLE-ABS-KEY (internet*)) OR (TITLE-ABS-KEY (web)) OR (TITLE-ABS-KEY (mobile*)) OR (TITLE-ABS-KEY (smartphone*)) OR (TITLE-ABS-KEY ({web-based})) OR (TITLE-ABS-KEY (ehealth)) OR (TITLE-ABS-KEY ({e-health})) OR (TITLE-ABS-KEY (mhealth)) OR (TITLE-ABS-KEY ({m-health})) OR (TITLE-ABS-KEY ({e-therapy})) OR (TITLE-ABS-KEY (telemental)) OR (TITLE-ABS-KEY (icbt)) OR (TITLE-ABS-KEY ({e-mail})) OR

(TITLE-ABS-KEY (email)) OR (TITLE-ABS-KEY (sms)) OR (TITLE-ABS-KEY ("text message*")) OR (TITLE-ABS-KEY ({text-message})) OR (TITLE-ABS-KEY (messaging)) OR (TITLE-ABS-KEY (ccbt)) OR (TITLE-ABS-KEY (chat*))) AND ((TITLE-ABS-KEY (depressi*)) OR (TITLE-ABS-KEY (dysthymi*)) OR (TITLE-ABS-KEY ("Premenstrual dysphoric disorder")) OR (TITLE-ABS-KEY ("mood disorder*")) OR (TITLE-ABS-KEY ("affective disorder*")) OR (TITLE-ABS-KEY (mdd)) OR (TITLE-ABS-KEY (anxiety)) OR (TITLE-ABS-KEY (anxious)) OR (TITLE-ABS-KEY (panic*)) OR (TITLE-ABS-KEY (phobi*)) OR (TITLE-ABS-KEY (gad)) OR (TITLE-ABS-KEY (fear)) OR (TITLE-ABS-KEY (agoraphobi*))) AND ((TITLE-ABS-KEY ("randomized controlled trial")) OR (TITLE-ABS-KEY ("controlled clinical trial")) OR (TITLE-ABS-KEY (randomized)) OR (TITLE-ABS-KEY (randomised)) OR (TITLE-ABS-KEY (randomly)) OR (TITLE-ABS-KEY (trial)) OR (TITLE-ABS-KEY (rct)))

5. PsycINFO via APA PsycNET

((((**IndexTermsFilt**: ("Relapse Prevention")))) *OR*(((**IndexTermsFilt**: ("Aftercare")))) *OR*(((**IndexTermsFilt**: ("Continuum Of Care")))) *OR*(((**title**: (aftercare))) *OR* ((**abstract**: (aftercare)))) *OR* (((**title**: (after-care))) *OR* ((**abstract**: (after-care)))) *OR* (((**title**: (after-treatment*))) *OR*((**abstract**: (after-treatment*)))) *OR* (((**title**: ("after treatment*"))) *OR* ((**abstract**: ("after treatment*")))) *OR* (((**title**: ("relapse prevention"))) *OR* ((**abstract**: ("relapse prevention")))) *OR* (((**title**: ("follow-up intervention"))) *OR* ((**abstract**: ("follow-up intervention")))) *OR* (((**title**: (post-treatment*))) *OR*((**abstract**: (post-treatment*)))) *OR* (((**title**: ("post treatment*"))) *OR* ((**abstract**: ("post treatment*")))) *OR* (((**title**: ("maintenance treatment*"))) *OR*((**abstract**: ("maintenance treatment*")))) *OR*(((**title**: ("continuation treatment*"))) *OR* ((**abstract**: ("continuation treatment*")))) *OR* (((**title**: ("post discharge"))) *OR* ((**abstract**: ("post discharge")))) *OR* (((**title**: ("tertiary prevention"))) *OR* ((**abstract**: ("tertiary prevention")))) *OR* (((**title**: ("secondary prevention"))) *OR* ((**abstract**: ("secondary prevention")))) *OR* (((**title**: (post-discharge))) *OR*((**abstract**: (post-discharge)))) *OR* (((**title**: ("continuity of care"))) *OR* ((**abstract**: ("continuity of care"))))) *AND* ((((**IndexTermsFilt**: ("Telemedicine")) *OR* (**IndexTermsFilt**: ("Teleconferencing")) *OR* (**IndexTermsFilt**: ("Online Therapy")) *OR* (**IndexTermsFilt**: ("Teleconsultation")) *OR* (**IndexTermsFilt**: ("Telepsychiatry")) *OR* (**IndexTermsFilt**: ("Telepsychology")) *OR* (**IndexTermsFilt**: ("Telerehabilitation")))) *OR* (((**IndexTermsFilt**: ("Computer Assisted Therapy")))) *OR*(((**IndexTermsFilt**: ("Internet")))) *OR*(((**IndexTermsFilt**: ("Mobile Health")))) *OR*(((**IndexTermsFilt**: ("Digital Interventions")))) *OR*(((**title**: (online*))) *OR* ((**abstract**: (online*)))) *OR*(((**title**: (computer*))) *OR* ((**abstract**: (computer*)))) *OR* (((**title**: (internet*))) *OR* ((**abstract**: (internet*)))) *OR* (((**title**: (web))) *OR* ((**abstract**: (web)))) *OR* (((**title**: (mobile*))) *OR* ((**abstract**: (mobile*)))) *OR* (((**title**: (smartphone*))) *OR*((**abstract**: (smartphone*)))) *OR* (((**title**: (web-based))) *OR* ((**abstract**: (web-based)))) *OR* (((**title**: (ehealth))) *OR* ((**abstract**: (ehealth)))) *OR* (((**title**: (e-health))) *OR* ((**abstract**: (e-health)))) *OR* (((**title**: (mhealth))) *OR* ((**abstract**: (mhealth)))) *OR* (((**title**: (m-health))) *OR* ((**abstract**: (m-health)))) *OR*(((**title**: (e-therapy))) *OR* ((**abstract**: (e-therapy)))) *OR* (((**title**: (telemental))) *OR* ((**abstract**: (telemental)))) *OR* (((**title**: (ICBT))) *OR* ((**abstract**: (ICBT)))) *OR* (((**title**: (e-mail))) *OR* ((**abstract**: (e-mail)))) *OR* (((**title**: (email))) *OR* ((**abstract**: (email)))) *OR* (((**title**: (SMS))) *OR* ((**abstract**: (SMS)))) *OR* (((**title**: ("text message*"))) *OR*((**abstract**: ("text message*")))) *OR* (((**title**: (text-message*))) *OR* ((**abstract**: (text-message*)))) *OR*(((**title**: (messaging))) *OR* ((**abstract**: (messaging)))) *OR* (((**title**: (CCBT))) *OR*((**abstract**: (CCBT)))) *OR* (((**title**: (chat*))) *OR*((**abstract**: (chat*))))) *AND* ((((**IndexTermsFilt**: ("Depression (Emotion)")))) *OR* (((**IndexTermsFilt**: ("Anxiety")))) *OR* (((**IndexTermsFilt**: ("Anxiety Disorders")) *OR* (**IndexTermsFilt**: ("Castration Anxiety")) *OR* (**IndexTermsFilt**: ("Death Anxiety")) *OR* (**IndexTermsFilt**: ("Generalized Anxiety Disorder")) *OR* (**IndexTermsFilt**: ("Obsessive Compulsive Disorder")) *OR* (**IndexTermsFilt**: ("Panic Attack")) *OR* (**IndexTermsFilt**: ("Panic Disorder")) *OR* (**IndexTermsFilt**: ("Phobias")) *OR*(**IndexTermsFilt**: ("Separation Anxiety

Disorder")) *OR* (**IndexTermsFilt**: ("Trichotillomania")))) *OR*(((**IndexTermsFilt**: ("Major Depression")) *OR*(**IndexTermsFilt**: ("Anaclitic Depression")) *OR*(**IndexTermsFilt**: ("Dysthymic Disorder")) *OR*(**IndexTermsFilt**: ("Endogenous Depression")) *OR*(**IndexTermsFilt**: ("Late Life Depression")) *OR*(**IndexTermsFilt**: ("Postpartum Depression")) *OR*(**IndexTermsFilt**: ("Reactive Depression")) *OR*(**IndexTermsFilt**: ("Recurrent Depression")) *OR*(**IndexTermsFilt**: ("Treatment Resistant Depression")))) *OR* (((**IndexTermsFilt**: ("Premenstrual Dysphoric Disorder")))) *OR*(((**IndexTermsFilt**: ("Fear")))) *OR* (((**title**: (depressi*))) *OR* ((**abstract**: (depressi*)))) *OR*(((**title**: (dysthymi*))) *OR* ((**abstract**: (dysthymi*)))) *OR* (((**title**: ("Premenstrual dysphoric disorder"))) *OR* ((**abstract**: ("Premenstrual dysphoric disorder")))) *OR* (((**title**: ("mood disorder*"))) *OR*((**abstract**: ("mood disorder*")))) *OR* (((**title**: ("affective disorder*"))) *OR* ((**abstract**: ("affective disorder*")))) *OR* (((**title**: (MDD))) *OR* ((**abstract**: (MDD)))) *OR* (((**title**: (anxiety))) *OR* ((**abstract**: (anxiety)))) *OR* (((**title**: (anxious))) *OR* ((**abstract**: (anxious)))) *OR* (((**title**: (panic*))) *OR* ((**abstract**: (panic*)))) *OR* (((**title**: (phobi*))) *OR* ((**abstract**: (phobi*)))) *OR* (((**title**: (GAD))) *OR* ((**abstract**: (GAD)))) *OR* (((**title**: (fear))) *OR* ((**abstract**: (fear)))) *OR* (((**title**: (agoraphobi*))) *OR* ((**abstract**: (agoraphobi*))))) *AND* ((((**title**: ("randomized controlled trial"))) *OR* ((**abstract**: ("randomized controlled trial"))) *OR* ((**Keywords**: ("randomized controlled trial")))) *OR* (((**title**: ("controlled clinical trial"))) *OR* ((**abstract**: ("controlled clinical trial"))) *OR* ((**Keywords**: ("controlled clinical trial")))) *OR*(((**title**: (randomized))) *OR* ((**abstract**: (randomized)))) *OR* (((**title**: (randomised))) *OR*((**abstract**: (randomised)))) *OR* (((**title**: (randomly))) *OR* ((**abstract**: (randomly)))) *OR*(((**title**: (trial))) *OR* ((**abstract**: (trial)))) *OR* (((**title**: (RCT))) *OR* ((**abstract**: (RCT)))))

6. PsycARTICLES via APA PsycNET

((((**IndexTermsFilt**: ("Relapse Prevention")))) *OR*(((**IndexTermsFilt**: ("Aftercare")))) *OR*(((**IndexTermsFilt**: ("Continuum Of Care")))) *OR*(((**title**: (aftercare))) *OR* ((**abstract**: (aftercare)))) *OR* (((**title**: (after-care))) *OR* ((**abstract**: (after-care)))) *OR* (((**title**: (after-treatment*))) *OR*((**abstract**: (after-treatment*)))) *OR* (((**title**: ("after treatment*"))) *OR* ((**abstract**: ("after treatment*")))) *OR* (((**title**: ("relapse prevention"))) *OR* ((**abstract**: ("relapse prevention")))) *OR* (((**title**: ("follow-up intervention"))) *OR* ((**abstract**: ("follow-up intervention")))) *OR* (((**title**: (post-treatment*))) *OR*((**abstract**: (post-treatment*)))) *OR* (((**title**: ("post treatment*"))) *OR* ((**abstract**: ("post treatment*")))) *OR* (((**title**: ("maintenance treatment*"))) *OR*((**abstract**: ("maintenance treatment*")))) *OR*(((**title**: ("continuation treatment*"))) *OR*((**abstract**: ("continuation treatment*")))) *OR*(((**title**: ("post discharge"))) *OR* ((**abstract**: ("post discharge")))) *OR* (((**title**: ("tertiary prevention"))) *OR* ((**abstract**: ("tertiary prevention")))) *OR* (((**title**: ("secondary prevention"))) *OR* ((**abstract**: ("secondary prevention")))) *OR* (((**title**: (post-discharge))) *OR* ((**abstract**: (post-discharge)))) *OR*(((**title**: ("continuity of care"))) *OR* ((**abstract**: ("continuity of care"))))) *AND* ((((**IndexTermsFilt**: ("Telemedicine")) *OR* (**IndexTermsFilt**: ("Teleconferencing")) *OR* (**IndexTermsFilt**: ("Online Therapy")) *OR* (**IndexTermsFilt**: ("Teleconsultation")) *OR* (**IndexTermsFilt**: ("Telepsychiatry")) *OR* (**IndexTermsFilt**: ("Telepsychology")) *OR* (**IndexTermsFilt**: ("Telerehabilitation")))) *OR* (((**IndexTermsFilt**: ("Computer Assisted Therapy")))) *OR*(((**IndexTermsFilt**: ("Internet")))) *OR*(((**IndexTermsFilt**: ("Mobile Health")))) *OR*(((**IndexTermsFilt**: ("Digital Interventions")))) *OR*(((**title**: (online*))) *OR* ((**abstract**: (online*)))) *OR*(((**title**: (computer*))) *OR* ((**abstract**: (computer*)))) *OR* (((**title**: (internet*))) *OR*((**abstract**: (internet*)))) *OR* (((**title**: (web))) *OR*((**abstract**: (web)))) *OR* (((**title**: (mobile*))) *OR*((**abstract**: (mobile*)))) *OR* (((**title**: (smartphone*))) *OR* ((**abstract**: (smartphone*)))) *OR* (((**title**: (web-based))) *OR* ((**abstract**: (web-based)))) *OR*(((**title**: (ehealth))) *OR* ((**abstract**: (ehealth)))) *OR*(((**title**: (e-health))) *OR* ((**abstract**: (e-health)))) *OR*(((**title**: (mhealth))) *OR* ((**abstract**: (mhealth)))) *OR*(((**title**: (m-health))) *OR* ((**abstract**: (m-health)))) *OR* (((**title**: (e-therapy))) *OR* ((**abstract**: (e-therapy)))) *OR* (((**title**:

(telemental))) *OR*((**abstract**: (telemental)))) *OR* (((**title**: (ICBT))) *OR*((**abstract**: (ICBT)))) *OR* (((**title**: (e-mail))) *OR*((**abstract**: (e-mail)))) *OR* (((**title**: (email))) *OR*((**abstract**: (email)))) *OR* (((**title**: (SMS))) *OR*((**abstract**: (SMS)))) *OR* (((**title**: ("text message*"))) *OR* ((**abstract**: ("text message*")))) *OR* (((**title**: (text-message*))) *OR* ((**abstract**: (text-message*)))) *OR* (((**title**: (messaging))) *OR*((**abstract**: (messaging)))) *OR* (((**title**: (CCBT))) *OR* ((**abstract**: (CCBT)))) *OR* (((**title**: (chat*))) *OR*((**abstract**: (chat*))))) *AND* ((((**IndexTermsFilt**: ("Depression (Emotion)")))) *OR* (((**IndexTermsFilt**: ("Anxiety")))) *OR* (((**IndexTermsFilt**: ("Anxiety Disorders")) *OR* (**IndexTermsFilt**: ("Castration Anxiety")) *OR* (**IndexTermsFilt**: ("Death Anxiety")) *OR* (**IndexTermsFilt**: ("Generalized Anxiety Disorder")) *OR* (**IndexTermsFilt**: ("Obsessive Compulsive Disorder")) *OR* (**IndexTermsFilt**: ("Panic Attack")) *OR* (**IndexTermsFilt**: ("Panic Disorder")) *OR* (**IndexTermsFilt**: ("Phobias")) *OR*(**IndexTermsFilt**: ("Separation Anxiety Disorder")) *OR* (**IndexTermsFilt**: ("Trichotillomania")))) *OR*(((**IndexTermsFilt**: ("Major Depression")) *OR*(**IndexTermsFilt**: ("Anaclitic Depression")) *OR*(**IndexTermsFilt**: ("Dysthymic Disorder")) *OR*(**IndexTermsFilt**: ("Endogenous Depression")) *OR*(**IndexTermsFilt**: ("Late Life Depression")) *OR*(**IndexTermsFilt**: ("Postpartum Depression")) *OR*(**IndexTermsFilt**: ("Reactive Depression")) *OR*(**IndexTermsFilt**: ("Recurrent Depression")) *OR*(**IndexTermsFilt**: ("Treatment Resistant Depression")))) *OR* (((**IndexTermsFilt**: ("Premenstrual Dysphoric Disorder")))) *OR*(((**IndexTermsFilt**: ("Fear")))) *OR* (((**title**: (depressi*))) *OR* ((**abstract**: (depressi*)))) *OR*(((**title**: (dysthymi*))) *OR* ((**abstract**: (dysthymi*)))) *OR* (((**title**: ("Premenstrual dysphoric disorder"))) *OR* ((**abstract**: ("Premenstrual dysphoric disorder")))) *OR* (((**title**: ("mood disorder*"))) *OR*((**abstract**: ("mood disorder*")))) *OR* (((**title**: ("affective disorder*"))) *OR* ((**abstract**: ("affective disorder*")))) *OR* (((**title**: (MDD))) *OR* ((**abstract**: (MDD)))) *OR* (((**title**: (anxiety))) *OR* ((**abstract**: (anxiety)))) *OR* (((**title**: (anxious))) *OR* ((**abstract**: (anxious)))) *OR* (((**title**: (panic*))) *OR* ((**abstract**: (panic*)))) *OR* (((**title**: (phobi*))) *OR* ((**abstract**: (phobi*)))) *OR* (((**title**: (GAD))) *OR* ((**abstract**: (GAD)))) *OR* (((**title**: (fear))) *OR* ((**abstract**: (fear)))) *OR* (((**title**: (agoraphobi*))) *OR* ((**abstract**: (agoraphobi*))))) *AND* ((((**title**: ("randomized controlled trial"))) *OR* ((**abstract**: ("randomized controlled trial"))) *OR* ((**Keywords**: ("randomized controlled trial")))) *OR* (((**title**: ("controlled clinical trial"))) *OR* ((**abstract**: ("controlled clinical trial"))) *OR* ((**Keywords**: ("controlled clinical trial")))) *OR*(((**title**: (randomized))) *OR* ((**abstract**: (randomized)))) *OR* (((**title**: (randomised))) *OR*((**abstract**: (randomised)))) *OR* (((**title**: (randomly))) *OR* ((**abstract**: (randomly)))) *OR*(((**title**: (trial))) *OR* ((**abstract**: (trial)))) *OR* (((**title**: (RCT))) *OR* ((**abstract**: (RCT)))))

7. PsycEXTRA via APA PsycNET

((((**IndexTermsFilt**: ("Relapse Prevention")))) *OR* (((**IndexTermsFilt**: ("Aftercare")))) *OR* (((**IndexTermsFilt**: ("Continuum Of Care")))) *OR* (((**title**: (aftercare))) *OR* ((**abstract**: (aftercare)))) *OR* (((**title**: (after-care))) *OR* ((**abstract**: (after-care)))) *OR* (((**title**: (after-treatment*))) *OR* ((**abstract**: (after-treatment*)))) *OR*(((**title**: ("after treatment*"))) *OR* ((**abstract**: ("after treatment*")))) *OR* (((**title**: ("relapse prevention"))) *OR* ((**abstract**: ("relapse prevention")))) *OR* (((**title**: ("follow-up intervention"))) *OR* ((**abstract**: ("follow-up intervention")))) *OR* (((**title**: (post-treatment*))) *OR* ((**abstract**: (post-treatment*)))) *OR* (((**title**: ("post treatment*"))) *OR*((**abstract**: ("post treatment*")))) *OR* (((**title**: ("maintenance treatment*"))) *OR*((**abstract**: ("maintenance treatment*")))) *OR* (((**title**: ("continuation treatment*"))) *OR* ((**abstract**: ("continuation treatment*")))) *OR* (((**title**: ("post discharge"))) *OR*((**abstract**: ("post discharge")))) *OR* (((**title**: ("tertiary prevention"))) *OR* ((**abstract**: ("tertiary prevention")))) *OR* (((**title**: ("secondary prevention"))) *OR* ((**abstract**: ("secondary prevention")))) *OR* (((**title**: (post-discharge))) *OR* ((**abstract**: (post-discharge)))) *OR* (((**title**: ("continuity of care"))) *OR* ((**abstract**:

("continuity of care"))))) *AND* ((((**IndexTermsFilt**: ("Telemedicine")) *OR* (**IndexTermsFilt**: ("Teleconferencing")) *OR* (**IndexTermsFilt**: ("Online Therapy")) *OR*(**IndexTermsFilt**: ("Teleconsultation")) *OR* (**IndexTermsFilt**: ("Telepsychiatry")) *OR*(**IndexTermsFilt**: ("Telepsychology")) *OR* (**IndexTermsFilt**: ("Telerehabilitation")))) *OR* (((**IndexTermsFilt**: ("Computer Assisted Therapy")))) *OR* (((**IndexTermsFilt**: ("Internet")))) *OR* (((**IndexTermsFilt**: ("Mobile Health")))) *OR* (((**IndexTermsFilt**: ("Digital Interventions")))) *OR* (((**title**: (online*))) *OR* ((**abstract**: (online*)))) *OR*(((**title**: (computer*))) *OR* ((**abstract**: (computer*)))) *OR* (((**title**: (internet*))) *OR*((**abstract**: (internet*)))) *OR* (((**title**: (web))) *OR* ((**abstract**: (web)))) *OR* (((**title**: (mobile*))) *OR* ((**abstract**: (mobile*)))) *OR* (((**title**: (smartphone*))) *OR* ((**abstract**: (smartphone*)))) *OR* (((**title**: (web-based))) *OR* ((**abstract**: (web-based)))) *OR*(((**title**: (ehealth))) *OR* ((**abstract**: (ehealth)))) *OR* (((**title**: (e-health))) *OR* ((**abstract**: (e-health)))) *OR* (((**title**: (mhealth))) *OR* ((**abstract**: (mhealth)))) *OR* (((**title**: (m-health))) *OR* ((**abstract**: (m-health)))) *OR* (((**title**: (e-therapy))) *OR* ((**abstract**: (e-therapy)))) *OR* (((**title**: (telemental))) *OR* ((**abstract**: (telemental)))) *OR* (((**title**: (ICBT))) *OR* ((**abstract**: (ICBT)))) *OR* (((**title**: (e-mail))) *OR* ((**abstract**: (e-mail)))) *OR* (((**title**: (email))) *OR* ((**abstract**: (email)))) *OR* (((**title**: (SMS))) *OR* ((**abstract**: (SMS)))) *OR* (((**title**: ("text message*"))) *OR* ((**abstract**: ("text message*")))) *OR*(((**title**: (text-message*))) *OR* ((**abstract**: (text-message*)))) *OR* (((**title**: (messaging))) *OR* ((**abstract**: (messaging)))) *OR* (((**title**: (CCBT))) *OR* ((**abstract**: (CCBT)))) *OR* (((**title**: (chat*))) *OR* ((**abstract**: (chat*))))) *AND* ((((**IndexTermsFilt**: ("Depression (Emotion)")))) *OR* (((**IndexTermsFilt**: ("Anxiety")))) *OR*(((**IndexTermsFilt**: ("Anxiety Disorders")) *OR* (**IndexTermsFilt**: ("Castration Anxiety")) *OR* (**IndexTermsFilt**: ("Death Anxiety")) *OR* (**IndexTermsFilt**: ("Generalized Anxiety Disorder")) *OR* (**IndexTermsFilt**: ("Obsessive Compulsive Disorder")) *OR* (**IndexTermsFilt**: ("Panic Attack")) *OR* (**IndexTermsFilt**: ("Panic Disorder")) *OR* (**IndexTermsFilt**: ("Phobias")) *OR* (**IndexTermsFilt**: ("Separation Anxiety Disorder")) *OR* (**IndexTermsFilt**: ("Trichotillomania")))) *OR*(((**IndexTermsFilt**: ("Major Depression")) *OR* (**IndexTermsFilt**: ("Anaclitic Depression")) *OR* (**IndexTermsFilt**: ("Dysthymic Disorder")) *OR* (**IndexTermsFilt**: ("Endogenous Depression")) *OR* (**IndexTermsFilt**: ("Late Life Depression")) *OR*(**IndexTermsFilt**: ("Postpartum Depression")) *OR* (**IndexTermsFilt**: ("Reactive Depression")) *OR* (**IndexTermsFilt**: ("Recurrent Depression")) *OR* (**IndexTermsFilt**: ("Treatment Resistant Depression")))) *OR* (((**IndexTermsFilt**: ("Premenstrual Dysphoric Disorder")))) *OR* (((**IndexTermsFilt**: ("Fear")))) *OR* (((**title**: (depressi*))) *OR* ((**abstract**: (depressi*)))) *OR* (((**title**: (dysthymi*))) *OR* ((**abstract**: (dysthymi*)))) *OR* (((**title**: ("Premenstrual dysphoric disorder"))) *OR* ((**abstract**: ("Premenstrual dysphoric disorder")))) *OR* (((**title**: ("mood disorder*"))) *OR* ((**abstract**: ("mood disorder*")))) *OR* (((**title**: ("affective disorder*"))) *OR* ((**abstract**: ("affective disorder*")))) *OR* (((**title**: (MDD))) *OR* ((**abstract**: (MDD)))) *OR* (((**title**: (anxiety))) *OR* ((**abstract**: (anxiety)))) *OR* (((**title**: (anxious))) *OR* ((**abstract**: (anxious)))) *OR*(((**title**: (panic*))) *OR* ((**abstract**: (panic*)))) *OR* (((**title**: (phobi*))) *OR* ((**abstract**: (phobi*)))) *OR* (((**title**: (GAD))) *OR* ((**abstract**: (GAD)))) *OR* (((**title**: (fear))) *OR*((**abstract**: (fear)))) *OR* (((**title**: (agoraphobi*))) *OR* ((**abstract**: (agoraphobi*)))))

8. PQDT Open

ti(aftercare OR after-treatment OR "after treatment*" OR "relapse prevention" OR post-treatment* OR "post treatment*" OR "post discharge" OR post-discharge OR "continuity of care") and if((online* OR computer* OR internet* OR web OR mobile* OR smartphone* OR web-based OR ehealth OR e-health OR mhealth OR m-health OR e-therapy OR telemental OR ICBT OR e-mail OR email OR SMS OR "text message*" OR text-message* OR messaging OR CCBT OR chat*) AND (depressi* OR dysthymi* OR "Premenstrual*

dysphoric disorder" OR "mood disorder" OR "affective disorder*" OR MDD OR anxiety OR anxious OR panic* OR phobi* OR GAD OR fear OR agoraphobi*))*

9. Open Access Theses and Dissertations
((title:(aftercare)) OR (title:(after-care)) OR (title:(after-treatment*)) OR (title:(after AND treatment*)) OR (title:(relapse AND prevention)) OR (title:(follow-up AND intervention)) OR (title:(post-treatment*)) OR (title:(post AND treatment*)) OR (title:(maintenance AND treatment*)) OR (title:(continuation AND treatment*)) OR (title:(post AND discharge)) OR (title:(tertiary AND prevention)) OR (title:(secondary AND prevention)) OR (title:(post-discharge)) OR (title:(continuity AND of AND care))) AND ((abstract:(online*)) OR (abstract:(computer*)) OR (abstract:(internet*)) OR (abstract:(web)) OR (abstract:(mobile*)) OR (abstract:(smartphone*)) OR (abstract:(ehealth)) OR (abstract:(mhealth)) OR (abstract:(e-therapy)) OR (abstract:(telemental)) OR (abstract:(ICBT)) OR (abstract:(email)) OR (abstract:(SMS)) OR (abstract:(text AND message*)) OR (abstract:(text-message*)) OR (abstract:(messaging)) OR (abstract:(CCBT)) OR (abstract:(chat*))) AND ((abstract:(depressi*)) OR (abstract:(dysthymi*)) OR (abstract:(premenstrual AND dysphoric AND disorder)) OR (abstract:(MDD)) OR (abstract:(anxiety)) OR (abstract:(anxious)) OR (abstract:(panic*)) OR (abstract:(phobi*)) OR (abstract:(GAD)) OR (abstract:(fear)) OR (abstract:(agoraphobi*)))

10. Open Grey

(abstract:aftercare OR after-care OR after-treatment* OR "after treatment*" OR "relapse prevention" OR "follow-up intervention" OR post-treatment* OR "post treatment*" OR "maintenance treatment*" OR "continuation treatment*" OR "post discharge" OR "tertiary prevention" OR "secondary prevention" OR post-discharge OR "continuity of care") AND (abstract:online* OR computer* OR internet* OR web OR mobile* OR smartphone* OR web-based OR ehealth OR e-health OR mhealth OR m-health OR e-therapy OR telemental OR ICBT OR e-mail OR email OR SMS OR "text message*" OR text-message* OR messaging OR CCBT OR chat*) AND (abstract:depressi* OR dysthymi* OR "Premenstrual dysphoric disorder" OR "mood disorder*" OR "affective disorder*" OR MDD OR anxiety OR anxious OR panic* OR phobi* OR GAD OR fear OR agoraphobi*)

11. CORDIS via cordis.europa.eu

('aftercare' OR 'after treatment' OR 'relapse prevention' OR 'follow-up intervention' OR 'post treatment' OR 'maintenance treatment' OR 'continuation treatment' OR 'post discharge' OR 'tertiary prevention' OR 'secondary prevention' OR 'continuity of care') AND ('online' OR 'computer' OR 'internet' OR 'web' OR 'mobile' OR 'smartphone' OR 'ehealth' OR 'mhealth' OR 'telemental' OR 'ICBT' OR 'email' OR 'SMS' OR 'text message' OR 'messaging' OR 'CCBT' OR 'chat') AND ('depression' OR 'dysthymia' OR 'Premenstrual dysphoric disorder' OR 'mood disorder' OR 'affective disorder' OR 'MDD' OR 'anxiety' OR 'anxious' OR 'panic' OR 'phobia' OR 'GAD' OR 'fear' OR 'agoraphobia' OR 'depressive' OR 'dysthymic')

12. ISRCTN Registry via BMC

In field: Text Search

(aftercare OR after-care OR after-treatment OR "after treatment" OR "relapse prevention" OR "follow-up intervention" OR post-treatment OR "post treatment" OR "maintenance treatment" OR "continuation treatment" OR "post discharge" OR "tertiary prevention" OR "secondary prevention" OR post-discharge OR "continuity of care") AND (online OR computer OR internet OR web OR mobile OR smartphone OR web-based OR ehealth OR e-health OR mhealth OR m-health OR e-therapy OR telemental OR ICBT OR e-mail OR email OR SMS OR "text message" OR text-message OR messaging

OR CCBT OR chat) AND (depression OR dysthymia OR "Premenstrual dysphoric disorder" OR "mood disorder" OR "affective disorder" OR MDD OR anxiety OR anxious OR panic OR phobia OR GAD OR fear OR agoraphobia OR depressive OR dysthymic)

In field: Trial status

Completed
