# Supplementary material for: Internet- and mobile-based aftercare and relapse prevention interventions for anxiety and depressive disorders: a systematic review
Source: Front Psychol. 2024 Dec 12;15:1474016. doi: 10.3389/fpsyg.2024.1474016 (PMC11670138; doi:10.3389/fpsyg.2024.1474016)
Supplement: Supplementary file 2 [file Data_Sheet_2.docx]

**Supplementary 2**

**Additional findings**

In addition to the primary outcomes of symptom severity and relapse rates, several studies reported findings that shed light on the broader impacts of digital interventions for depression. These additional outcomes encompass a range of physiological, cognitive, and psychosocial domains, providing a more comprehensive understanding of the interventions' effects.

The positive face-based Attentional Bias Modification (ABM) intervention not only reduced anxiety symptoms but also decreased the cortisol awakening response during follow-up (*p*=0.03) (Browning et al., 2012). This finding suggests that digital interventions may have measurable effects on biological stress markers. Furthermore, the same study reported an increase in positive attentional bias on a Word-based Visual probe task (*p*=0.002), indicating successful modification of cognitive processes. The results showed significant reductions in maladaptive emotion regulation and increases in resilience in the cognitive control training group (Hoorelbeke et al., 2015), but no significant effects on adaptive emotion regulation. Functional outcomes were addressed in the study by Vicent-Gil et al. (2022), which reported significant improvements in verbal memory, executive function, and overall functioning as measured by FAST for the INCREM group at 26 weeks (*p*=0.041). Sleep parameters were examined by Aggestrup et al. (2023). The Circadian Reinforcement Therapy (CRT) group demonstrated better sleep quality (*p*=0.04), earlier sleep onset (*p*=0.009), and longer sleep duration (*p*=0.005) compared to the treatment as usual group. Additionally, the results showed lower day-to-day variability in mood and sleep parameters in the CRT group (all *p*<0.001), suggesting a stabilizing effect on circadian rhythms.

For individuals with comorbid alcohol use disorder, the findings showed significantly greater reduction in units per drinking day in the intervention group at 6 months, although this difference was not maintained at the 12-month follow-up (O’Reilly et al., 2019). The same study reported significantly greater reductions in perceived stress at 3 months, as measured by the PSS.

Rumination, a cognitive process often implicated in depression, was addressed by Zwerenz et al. (2017), who found lower rumination scores in the intervention group (B: -6.09, 95% CI: -11.19 to -1.00, *p*=0.019).

Patient satisfaction, an important aspect of intervention acceptability, was explored by Simon et al. (2011), who reported greater satisfaction in the intervention group, with 53% vs 33% reporting being "very satisfied" (*p*=0.004). Similarly, Hunkeler et al. (2012) found improvements in mental health-related quality of life, as measured by the SF-36 mental health scores (*p*=0.002).
